# A tripartite survey of hyperparasitic fungi associated with ectoparasitic flies on bats (Mammalia: Chiroptera) in a neotropical cloud forest in Panama

**DOI:** 10.1051/parasite/2018017

**Published:** 2018-04-10

**Authors:** Melissa J. Walker, Annabel Dorrestein, Jasmin J. Camacho, Lauren A. Meckler, Kirk A. Silas, Thomas Hiller, Danny Haelewaters

**Affiliations:** 1 Department of Ecology, Environment and Evolution, La Trobe Institute for Molecular Science Bundoora, Victoria 3083 Australia; 2 Current address: Hawkesbury Institute for the Environment, Western Sydney University, Science Road, Richmond, New South Wales 2753 Australia; 3 Department of Animal Ecology, Utrecht University, Padualaan 8, 3584 CH Utrecht The Netherlands; 4 Department of Organismic and Evolutionary Biology, Harvard University, 26 Oxford Street, Cambridge, Massachusetts 02138 USA; 5 Department of Psychological and Brain Sciences, University of Delaware, 105 The Green, Newark, Delaware 19716 USA; 6 Painted Sky Road, Reading, Pennsylvania 19606 USA; 7 Institute of Evolutionary Ecology and Conservation Genomics, University of Ulm, Albert-Einstein Allee 11, 89081 Ulm Germany; 8 Smithsonian Tropical Research Institute, Apartado Postal 0843-03092, Balboa Panama; 9 Herbario UCH, Universidad Autónoma de Chiriquí, Apartado Postal 0427, David Panama

**Keywords:** Bat flies, Diptera, Ectoparasites, Hyperparasites, Laboulbeniales, Sequence-based identification of fungi

## Abstract

The Darién province in eastern Panama is one of the most unexplored and biodiverse regions in the world. The Chucantí Nature Reserve, in Serranía de Majé, consists of a diverse tropical cloud forest ecosystem. The aim of this research was to explore and study host associations of a tripartite system of bats, ectoparasitic flies on bats (Diptera, Streblidae), and ectoparasitic fungi (Ascomycota, Laboulbeniales) that use bat flies as hosts. We captured bats at Chucantí, screened each bat for presence of bat flies, and screened collected bat flies for presence of Laboulbeniales. We mistnetted for 68 mistnet hours and captured 227 bats representing 17 species. We captured *Micronycteris schmidtorum*, a species previously unreported in Darién. In addition, we encountered the rarely collected *Platyrrhinus dorsalis*, representing the westernmost report for this species. Of all captured bats, 148 carried bat flies (65%). The number of sampled bat flies was 437, representing 16 species. One species represents a new country record (*Trichobius anducei*) and five species represent first reports for Darién (*Basilia anceps*, *Anatrichobius scorzai*, *Nycterophilia parnelli*, *T. johnsonae*, *T. parasiticus*). All 74 bat fly species currently reported in Panama are presented in tabulated form. Of all screened bat flies, 30 bore Laboulbeniales fungi (7%). Based on both morphology and large ribosomal subunit (LSU) sequence data, we delimited 7 species of Laboulbeniales: *Gloeandromyces nycteribiidarum* (newly reported for Panama), *G. pageanus*, *G. streblae*, *Nycteromyces streblidinus*, and 3 undescribed species. Of the 30 infected flies, 21 were *Trichobius joblingi*. This species was the only host on which we observed double infections of Laboulbeniales.

## Abstract (fr)

La province de Darién dans l’est du Panama est l’une des régions les plus inexplorées et les plus riches en biodiversité au monde. La réserve naturelle du Chucantí, à Serranía de Majé, est constituée d’un écosystème forestier tropical varié. Le but de cette recherche était d’explorer et d’étudier les associations hôtes d’un système tripartite de chauves-souris, de mouches ectoparasites sur chauves-souris (Diptera, Streblidae) et de champignons ectoparasites (Ascomycota, Laboulbeniales) utilisant des diptères de chauves-souris comme hôtes. Nous avons capturé des chauves-souris à Chucantí, criblé chaque chauve-souris pour la présence de diptères, et criblé les diptères recueillis pour la présence de Laboulbeniales. Nous avons utilisé des filets japonais pendant 68 heures et avons capturé 227 chauves-souris représentant 17 espèces. Nous avons capturé *Micronycteris schmidtorum*, une espèce précédemment non rapportée de Darién. En outre, nous avons rencontré l’espèce rarement collectée *Platyrrhinus dorsalis*, ce qui représente la mention la plus occidentale de cette espèce. De toutes les chauves-souris capturées, 148 portaient des diptères (65%). Le nombre de diptères échantillonnés était de 437, représentant 16 espèces. Une espèce représente une nouvelle mention pour ce pays (*Trichobius anducei*) et cinq espèces représentent les premières mentions pour Darién (*Basilia anceps*, *Anatrichobius scorzai*, *Nycterophilia parnelli*, *T. johnsonae*, *T. parasiticus*). Les 74 espèces de diptères de chauves-souris actuellement signalées au Panama sont présentées sous forme de tableau. Parmi tous les diptères examinés, 30 portaient des champignons Laboulbeniales (7%). Sur la base de la morphologie et des données des séquences d’ADNr LSU, nous avons délimité 7 espèces de Laboulbeniales : *Gloeandromyces nycteribiidarum* (nouvelle mention pour Panama), *G. pageanus*, *G. streblae*, *Nycteromyces streblidinus* et 3 espèces non décrites. Sur les 30 mouches infectées, 21 étaient des *Trichobius joblingi*. Cette espèce est le seul hôte sur lequel nous avons observé des doubles infections de Laboulbeniales.

## Introduction

Panamanian forests are under major risk of deforestation, threatening the associated biodiversity. The 2015 Global Forest Resources Assessment reports that 62.1% of Panama is forested; however, there was an annual change rate of −0.4% between 1990 and 2015 [23]. Recent efforts by various independent bodies and organizations have focused on securing existing forests, reforesting farm lands, and reporting on unidentified and known species endemic to the region. In recent years, many new species of plants, animals, and fungi have been reported in Panamanian forests. A Google Scholar search (on September 23, 2017) using the keywords “new species” and “Panama” resulted in 1,740 hits since 2017 alone. This collaborative management has benefited Panama’s native flora and fauna, while promoting the importance of rainforest conservation [61,81,82,96,97]. Although research efforts are steadfast, difficult terrain and political strife leaves many Panamanian locations isolated and difficult to explore.

One such location is the Darién province, in eastern Panama. Darién, host to the Darién National Park, is one of the most pristine habitats in Central America, and one of the most endemically biodiverse zones in the world. Dividing the Panamá and Darién provinces is the Serranía de Majé, a 60 km long, 404 ha mountain range with elevations from 600 to 1480 m a.s.l. [2,71]. The summit of Serranía de Majé is Cerro Chucantí, a diverse tropical cloud forest ecosystem [71] and host to the Chucantí Nature Reserve. However, the rough terrain, proximity to Colombian border, and isolation of Cerro Chucantí has thus far resulted in many species going undocumented.

Prior to private purchase, parts of Chucantí Nature Reserve (hereafter: Chucantí) were subject to logging, established as farmland, and severely under threat by agricultural and livestock activities [17,48,55]. While this threat is still evident in regions of the Serranía de Majé, much of Chucantí is recovering. This constructive reestablishment of the natural ecosystem, in conjunction with researchers obtaining supported access to the mountain, has spurred greater insight into the environment of this area. Accordingly, we documented bat species richness and abundance at three altitudes in Chucantí. We screened all captured bats for the presence of bat flies, which in turn were screened for the presence of Laboulbeniales fungi, with the aim of reporting biodiversity at different trophic levels (host–parasite–parasite) and studying host associations. Below, we briefly introduce the three levels of this hyperparasitic study system.

### Bats (Mammalia: Chiroptera)

Bats are the only mammals capable of sustained flight. They use echolocation and have a wide assortment of food sources, thereby providing important ecosystem services like insect predation and seed dispersal [54]. The New World leaf-nosed bats (family Phyllostomidae) are the most morphologically and ecologically diverse of all bats. They have evolved extraordinarily diverse faces, skulls, and teeth, adapted to many different food types, including insects, other vertebrates, blood, fruit, and nectar [26]. Bats also differ greatly in their roosting habits; roosts vary from more permanent and enclosed structures (caves, rock crevices, mines) to ephemeral and exposed structures (leaf tents, plant foliage) [53].

There are around 1,200 species of bats worldwide [9]. In Panama, a total of 118 bat species are documented, therefore representing the most diverse mammal group in the country [78]. Although these species reports are numerous, many of the results come from lowland research, leaving many highland Panamanian regions without mammal inventories; Chucantí is one of them [43,77]. Given the isolation of Chucantí, it is not surprising that only limited biodiversity reports from this area are available, and none dealing with bats. With a variety of morphological differences, dietary differentiation, and ecological requirements, bats are assumed to cohabitate and exploit the ecological diversity of the Chucantí cloud forests.

### Bat flies (Diptera: Hippoboscoidea: Streblidae and Nycteribiidae)

Bat flies are obligate blood-feeding ectoparasites of bats. They are generally assigned to two families: the monophyletic Nycteribiidae with a mainly Palaearctic distribution, and the paraphyletic Streblidae that are most diverse in the Neotropics [18]. On their bat host, they show preferences for certain body areas like the furry body or the wing membranes [84]. Bat flies spend most of their life on their hosts, and female bat flies only leave to deposit 3rd instar larvae in the bat roost, which immediately pupate [16].

Bat flies are highly host specific [13,15]. Large-scale bat surveys in the field have led to this understanding. For example, in Paraguay, 2,893 captured bats yielded 2,467 bat flies, of which 87.1% were highly host specific. Bat social structure and bat roosting behavior are important contributors to population dynamics of these parasites [74]. The majority of the neotropical Streblidae are parasites of Phyllostomidae, the highly diverse leaf-nosed bats [15,39,93,94]. Currently, a total of 73 bat fly species are known to occur in Panama [27,39,94]. Of these, a majority belong to Streblidae (66 species).

As parasites comprise a crucial part of bat ecology [95], sampling bat flies together with biological data of the host individuals can provide valuable information on animal health [56]. Bat flies, too, can serve as hosts to smaller biota, such as the enigmatic, microscopic Laboulbeniales fungi.

### Laboulbeniales (Fungi: Ascomycota: Laboulbeniomycetes)

The order Laboulbeniales consists of microscopic ectosymbionts of myriad arthropods. As an exception among related groups of fungi, they do not grow hyphae but form a “reduced hyphal system” (*thallus*, plural *thalli*) formed by a predetermined number of mitotic divisions. A thallus typically consists of 3 main parts: a receptacle, which attaches to the host; a perithecium, the spore-forming structure, or multiple perithecia; and appendages with antheridia that produce spermatia. Laboulbeniales are usually host specific, often with a one-on-one relationship. The inverse can also be true, when one species or morphologically similar species of Laboulbeniales are associated with completely unrelated hosts. In this case, phylogenetically unrelated hosts inhabit the same microhabitat, providing opportunities for fungal ectoparasites to transmit to atypical hosts [*sensu* 12]. As different species of bat flies can occur on a single bat, the bat as a whole serves as a microhabitat, and thus we hypothesize that at least sometimes transmission between typical and atypical bat fly hosts will take place.

About 80% of described species of Laboulbeniales are found on Coleoptera, and only 10% on Diptera [92]. Laboulbeniales associated with flies belong to 8 genera, 3 of those are exclusive to bat flies: *Arthrorhynchus*, *Gloeandromyces*, and *Nycteromyces* [41]. Only the genera *Gloeandromyces* and *Nycteromyces* occur on neotropical bat flies. Thus far in Panama, only 3 species of Laboulbeniales have been reported on bat flies: *Gloeandromyces pageanus*, which was described from bat flies collected in Gamboa in the Canal Zone (Colón Province), *G. streblae*, and *Nycteromyces streblidinus* [42].

## Material and Methods

### Ethics and permits

All capture and sampling procedures were licensed and approved by the Smithsonian Tropical Research Institute (IACUC protocol: 2017-0102-2020-A5) and the Government of Panama (Ministerio de Ambiente de Panamá: SE/AH-2-16, SC/AH-1-17).

### Field sites

**Figure 1:**
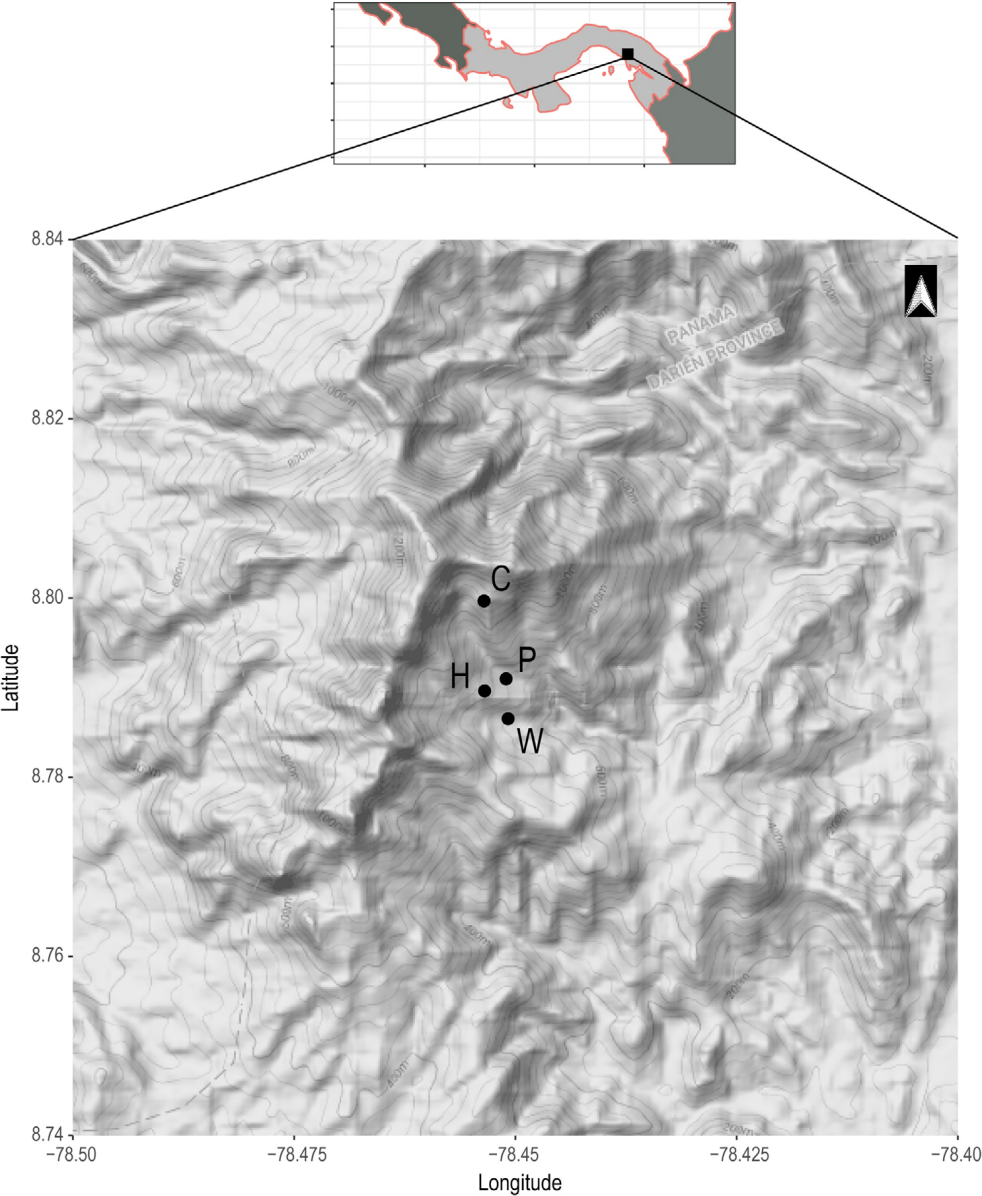
Geographical map of the studied area, with its location in Panama. The four sampled field sites at Chucantí Nature Reserve are indicated as follows: C = Camp Site, H = Helipad, P = Potrerito, and W = Waterfall.

We visited Chucantí (8.8046°N, 78.4595°W) from June 17 to 25, 2017 (rainy season). Chucantí is a large area of submontane forest surrounded by livestock pastures although still in contact with original vegetation. The reserve has premontane wet forests and tropical moist forests [*sensu* 45]. Within these forest types, secondary forest succession describes three of our field sites, defined by the degree of disturbance of the area. We visited one field site, an undisturbed primary forest, comparatively. Bats were captured at these four field sites at Chucantí over seven nights (Figure 1). (1) “Helipad” was a heavily disturbed area, approximately 25 m perpendicular to the Loop Trail, which circles around the research station towards the north. The site was felled during the time of our visit. As a result, the area was cleared of most vegetation, except for low grasses and some dispersed trees, and designated as early secondary succession. Close-by, towards the south of the field site, was an open field with high grasses. (2) “Waterfall” describes the field site with old-growth broadleaf characteristics presenting proliferation of secondary vegetation and loss of arboreal cover, adjacent to a river and waterfall, with flyways heavily disturbed by horses, construction workers, and staff of the nature reserve; young secondary succession. (3) “Potrerito”, located along a trail with the same name that runs from the research station to the Camp Site, was situated in an old-growth, broadleaf forest close to a river, which opened towards a recovering livestock pasture with tall grasses, frequented by horses; middle-aged secondary succession. (4) “Camp Site” was located on the trail towards the summit of Chucantí, with many palm trees, oaks, fig trees, epiphytes, and bromeliads growing densely on tree branches and stems, and a few tall, giant trees. This site, to our knowledge, has not been used as agricultural land, and is the least disturbed of our sites, thus classified as primary forest.

### Capture of bats and collection of bat flies

Bats were captured using three to four 6-m ground level mistnets (36-mm mesh, 4 shelves, Avinet, Portland, ME, USA). Mistnets were positioned over existing trails, which we assessed to be used by bats as flight paths [73], including streams and close to water bodies. The nets were usually open from sunset to around 11 pm and examined every 10 min. Bats were disentangled quickly and kept in clean, soft cotton bags until processing. Bats were released at the vicinity of the capture site immediately after processing. Most nectarivorous and frugivorous bats were fed with sugar water prior to release.

Captured bats were identified on site using dichotomous keys [44,87]. For each bat, the following characteristics were noted: species, sex, age (juvenile, subadult, adult), reproductive status (pregnant, lactating, post-lactating, scrotal, non-reproductive), forearm length (in mm), body mass (in g), and whether bat flies were collected. We also noted whether a wing punch and/or photographs were taken. When abnormalities such as abscesses, wounds, or broken fingers or teeth were observed, comments were added for each respective bat. Bat taxonomy follows Simmons [79].

In order to remove ectoparasitic bat flies, we used a paintbrush to cover the flies on the bat with 96% ethanol. Subsequently, the bat flies were taken using forceps. Some bat flies were collected using the forceps only or by hand. Two types of forceps were used: rigid Swiss Style Forceps #5 with superfine tip (BioQuip #4535, Rancho Dominguez, CA, USA) and Featherweight Forceps with narrow tip (BioQuip #4748). Preservation and long-term storage of bat flies was in 96% ethanol in separate vials (one vial per bat host). Identification of bat flies to species level was based on published keys [29–37,93,94] and complementary publications [14,66]. Voucher specimens are deposited at the following locations: Museo de Peces de Agua e Invertebrados, David, Panamá (MUPADI) and Naturalis Biodiversity Center, Leiden, The Netherlands (RMNH).

### Collection and identification of Laboulbeniales

Bat flies were examined under a Zeiss Stemi 508 stereomicroscope (Thornwood, New York) for the presence of Laboulbeniales thalli. Individual thalli were removed from the host at the point of attachment (foot or haustorium) using Minuten Pins (BioQuip #1208SA, Rancho Dominguez, CA, USA) inserted onto wooden rods. We prepared microscope slides onto which thalli were mounted in Amann solution [6], with modifications. We placed a droplet of Hoyer’s medium on the slide with the tip of a Minuten pin and deposited thalli in the droplet. The thalli were positioned onto the slide by placing them in a single row, each thallus in the tiniest droplet of Hoyer’s medium. We then placed a droplet of the Amann solution on the cover slip, and dropped it (droplet facing down) sideways onto the Hoyer’s medium. The cover slip was coated with transparent B-72 (Gaylord #AB72, Syracuse, NY, USA) or nail varnish. We viewed mounted specimens at 400x to 1000x magnification for identification using those publications with descriptions and illustrations of Laboulbeniales occurring on bat flies [42,85,86]. Voucher slides are deposited at Farlow Herbarium (FH; Harvard University, Cambridge, MA, USA) and Herbario de la Universidad Autónoma de Chiriquí (UCH; David, Panamá).

### DNA extraction, amplification, phylogenetic analysis

DNA was extracted from 1-4 Laboulbeniales thalli using a modified REPLI-g Single Cell Kit (Qiagen, Valencia, CA, USA) protocol [40]. Thalli were often manually cut in 2-3 parts (through the perithecium) using a #10 surgical blade on disposable Bard-Parker handle (Aspen Surgical, Caledonia, MI, USA) to ensure successful lysis. For the purpose of this survey, we only amplified the nuclear large ribosomal subunit (LSU) using primers LR0R (5’–ACCCGCTGAACTTAAGC–3’) and LR5 (5’–ATCCTGAGGGAAACTTC–3’). PCR reactions consisted of 13.3 μL of RedExtract Taq polymerase (Sigma-Aldrich, St. Louis, MO, USA), 2.5 μL of each 10 μM primer, 5.7 μL of H_2_O, and 1.0 μL of template DNA. All amplifications were done using a 2720 Thermal Cycler (Applied Biosystems, Foster City, CA, USA) with initial denaturation at 94 °C for 3:00 min; followed by 35 cycles of 94 °C for 1:00 min, 50 °C for 0:45 min, and 72 °C for 1:30 min; and final extension at 72 °C for 10:00 min. PCR products were purified using the QIAquick PCR Purification Kit (Qiagen) and subsequently sequenced at the Molecular Multi-User’s Lab at the Naos Marine Laboratories (Smithsonian Tropical Research Institute, Panama). We prepared 10 µL reactions with the same primers and 3.0 µL of purified PCR product. The sequencing reactions were performed using the Big Dye^®^ Terminator v3.1 Cycle Sequencing Kit (Life Technologies, Carlsbad, CA, USA). Generated sequences were assembled and edited in Sequencher 4.10.1 (Gene Codes Corporation, Ann Arbor, MI, USA). All sequences will be deposited in GenBank.

We constructed an LSU dataset of newly generated sequences and sequences downloaded from GenBank to assess species discrimination in the genus *Gloeandromyces*. Alignment of the LSU data matrix was done using Muscle v3.7 [21] on the Cipres Science Gateway, version 3.3 [65]. Maximum likelihood (ML) analysis was run using PAUP on XSEDE 4.0b [83], which is available on Cipres. The appropriate nucleotide substitution model was selected by considering the Akaike Information Criterion (AIC) in jModelTest 2.1 [11]. The general time reversible model (GTR) with estimation of invariant sites (+I) and the assumption of a gamma distribution with six rate categories (+G) gave the best scoring tree (-lnL = 2867.4857). ML was inferred under this model and bootstrap (BS) values were calculated with 1000 replicates.

### Diversity analyses

Further analyses of species diversity and network associations were done using the R language and environment for statistical computing [75]. A species accumulation curve was achieved using the ’vegan’ package [70]. In addition, all collected data were combined by building a network of associations between bats, bat flies, and Laboulbeniales. This visualization was done with the help of the ’bipartite’ package, using the plotweb function [19].

## Results

### Bat species richness and dominance

We mistnetted at 4 sites for 7 nights with a total netting effort of 68 mistnet hours (mnh), where 1 mnh equals a single 6 m-wide mistnet open for 1 hour (Table 1). We captured a total of 227 bats representing 17 species in 3 families (Table 2). All bat species captured at Chucantí are summarized in the Supplementary Material: Table S1, including the number of captured individuals, average forearm length (in mm), average body mass (in g), and photos taken in the field. The family Phyllostomidae was best represented with 14 species. The individuals of this family account for 95.15% of all captures. The most common species was *Carollia perspicillata* with 62.56% of all captures (*n* = 142), followed by *Artibeus jamaicensis* with 18.94% (*n* = 43). Seven species were represented in our dataset by a single individual: *Enchisthenes hartii*, *Lichonycteris obscura*, *Micronycteris microtis*, *M. schmidtorum*, *Platyrrhinus dorsalis*, *P. helleri*, and *Sturnira luisi*.

**Table 1:** Locality, coordinates and elevation (in m a.s.l.) of the mistnetting locations, together with the number of nights netted, the capturing effort (mnh), the number of bat species and bat individuals captured, and the number of bats/mnh.

Field site	Coordinates	Elevation	Nights	Capturing effort (mnh)	No. of species	No. of individuals	Bats/mnh
Helipad	N 08°47.378’ W078°27.209’	831	1	5.1	8	46	9
Waterfall	N 08°47.191’ W078°27.050’	681	2	25.7	7	77	3
Portrerito	N 08°47.459’ W078°27.062’	733	2	22.2	10	84	3.8
Camp Site	N 08°47.981’ W078°27.213’	1141	2	15	6	20	1.3
Total			7	68.0	17	227	5

**Table 2:** The number of individuals captured per species at each locality. Species that have not been documented in Darién before are shown in bold [*sensu* 43,62]. Conservation status and current population trend are given for each species, according to the IUCN Red List. Handley Jr. [43] referred to the following species by different names, following contemporaneous taxonomy: *Micronycteris microtis* (as *megalotis*), *Myotis riparius* (as *simus riparius*), *Platyrrhinus helleri* (as *Vampyrops helleri*), *Pteronotus gymnonotus* (as *suapurensis*), and *Sturnira luisi* (as *ludovici*).

Taxon	Helipad	Waterfall	Potrerito	Camp Site	Conservation	Population
PHYLLOSTOMIDAE						
* Artibeus jamaicensis*	14	7	13	9	Least Concern	Stable
* Artibeus lituratus*	0	7	3	1	Least Concern	Stable
* Carollia brevicauda*	1	0	1	2	Least Concern	Stable
* Carollia perspicillata*	24	59	57	2	Least Concern	Stable
* Desmodus rotundus*	2	0	1	0	Least Concern	Stable
* Enchisthenes hartii*	1	0	0	0	Least Concern	Unknown
* Glossophaga commissarisi*	1	1	0	0	Least Concern	Stable
* Lichonycteris obscura*	0	1	0	0	Least Concern	Unknown
* Micronycteris microtis*	0	1	0	0	Least Concern	Stable
* **Micronycteris schmidtorum***	0	0	1	0	Least Concern	Stable
* Platyrrhinus dorsalis*	0	0	0	1	Least Concern	Unknown
* Platyrrhinus helleri*	0	0	1	0	Least Concern	Stable
* Sturnira luisi*	1	0	0	0	Least Concern	Unknown
* Trachops cirrhosus*	0	1	2	1	Least Concern	Stable
MORMOOPIDAE						
* Pteronotus gymnonotus*	0	0	2	0	Least Concern	Stable
* Pteronotus parnellii*	2	0	2	0	Least Concern	Stable
VESPERTILIONIDAE						
* Myotis riparius*	0	0	1	4	Least Concern	Stable
Total	46	77	84	20		

**Figure 2:**
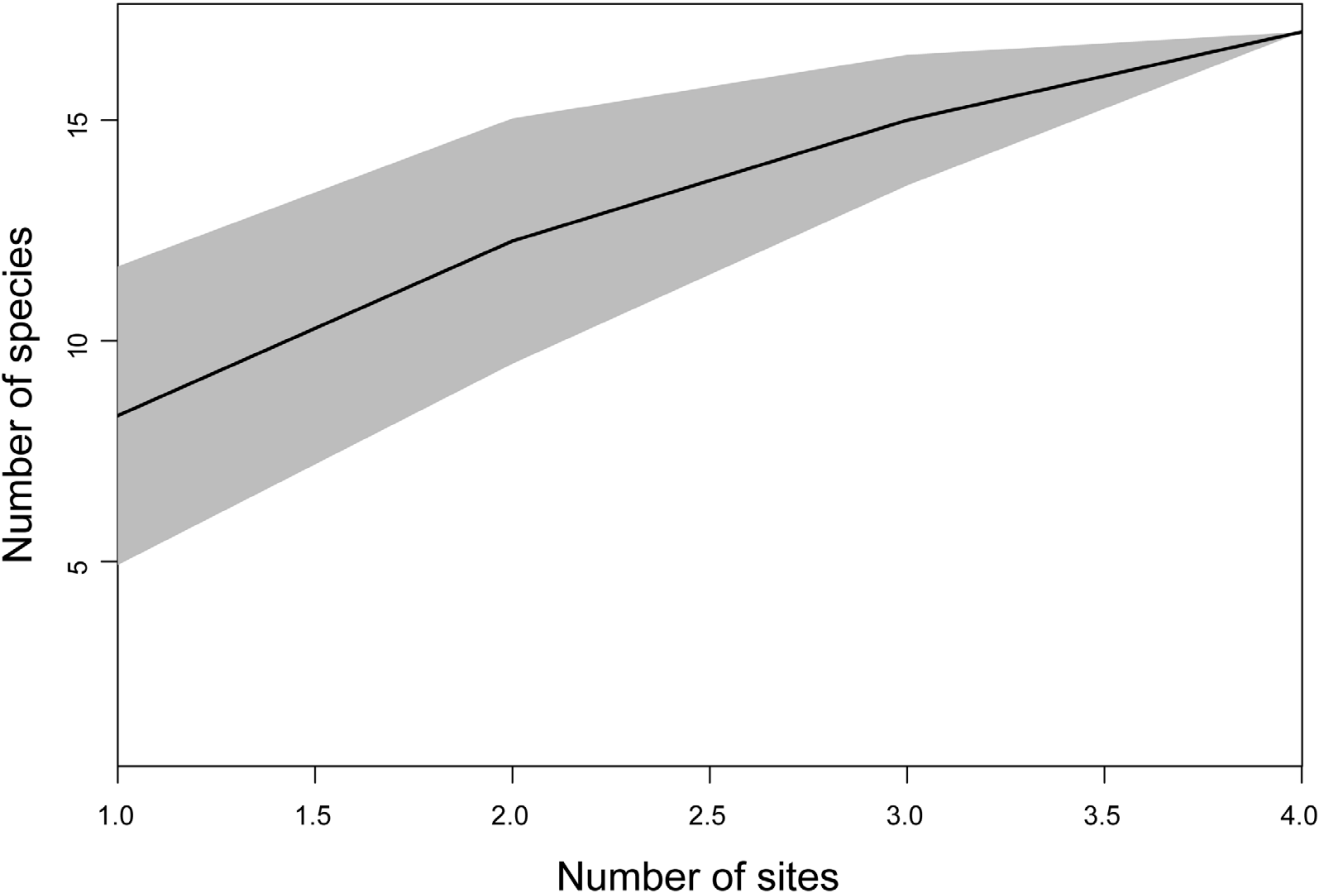
Species accumulation curve of the number of bat species captured at each site. The sites on the X-axis are randomized and cumulative. The area in grey represents the confidence interval.

**Figure 3:**
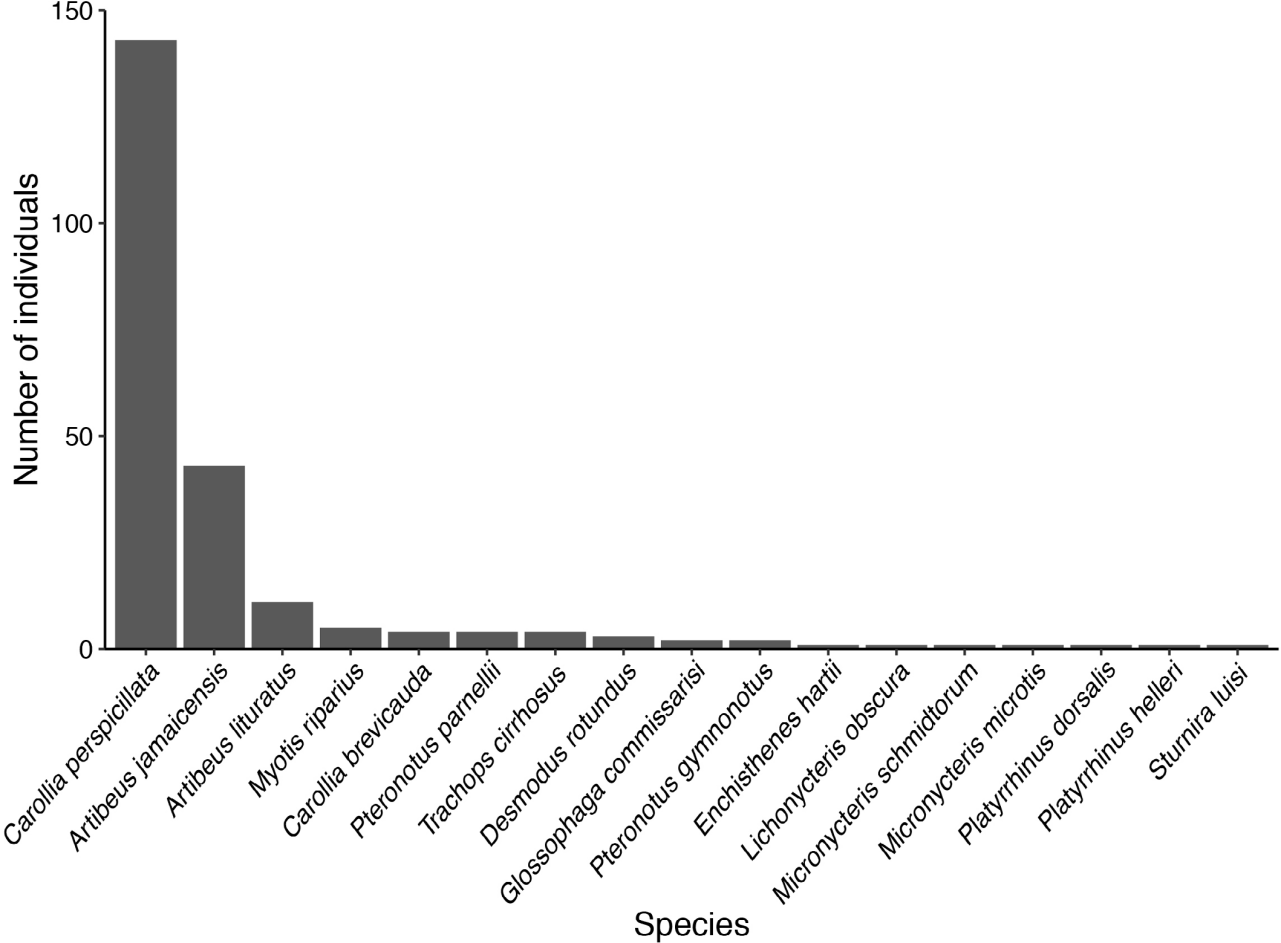
Number of individuals per bat species captured in Chucantí.

The highest number of bat species and bat individuals were captured at Potrerito. The lowest number of individuals and species were captured at the Camp Site (Table 2). To assess the completeness of our survey, we plotted the cumulative number of species against the number of sites we surveyed. The data were randomized to exclude the effects of the order of the sites on the X-axis. The graph in Figure 2 does not reach an asymptote, indicating that our survey did not cover the bat diversity present in the area. The relative abundance distribution in Figure 3 visualizes the number of rare and abundant species. The abundance of most of the species we captured is relatively low, with only two species, *A. jamaicensis* and especially *C. perspicillata*, being highly abundant.

### Bat flies and Laboulbeniales

Of the 227 bats we sampled at Chucantí, 148 carried bat flies (parasite prevalence of 65%). A total of 437 bat flies were collected, representing 16 species, of which 15 belong to the family Streblidae and one (*Basilia anceps*) to the family Nycteribiidae. Details are presented in Table 3. The 6 specimens of *Trichobius anducei*, all collected from *C. perspicillata* bats, represent the first report of this species for Panama. Five species had not yet been reported from Darién. These are *Anatrichobius scorzai*, *B. anceps*, *Nycterophilia parnelli*, *Trichobius johnsonae*, and *T. parasiticus*.

**Table 3:** List of bat fly species collected during this study, with indication of their bat host species and number of infected bats, the number of collected bat flies (N), and the specificity index (SI) = the percentage of total bat flies of a single species found on one host.

Bat host species (#)	Bat fly species	N	SI
*Artibeus jamaicensis* (43)	*Megistopoda aranea*	14	0.67
	*Paratrichobius longicrus*	1	1.00
	*Aspidoptera phyllostomatis*	8	0.89
*Artibeus lituratus* (11)	*Megistopoda aranea*	6	0.29
	*Aspidoptera phyllostomatis*	1	0.11
*Carollia brevicauda* (4)	Trichobius joblingi*	7	0.03
*Carollia perspicillata* (142)	*Speiseria ambigua*	43	0.96
	*Strebla guajiro*	22	1.00
	*Trichobius joblingi*	248	0.97
	*Trichobius anducei*	6	1.00
*Desmodus rotundus* (1)	*Trichobius parasiticus*	1	1.00
*Micronycteris schmidtorum* (1)	Trichobius joblingi*	1	0.00
*Myotis riparius* (5)	*Basilia anceps*	1	1.00
	*Anatrichobius scorzai*	3	1.00
*Pteronotus gymnonotus* (1)	*Trichobius johnsonae*	1	1.00
*Pteronotus parnellii* (4)	*Nycterophilia parnelli*	1	1.00
	*Trichobius yunkeri*	49	1.00
	Megistopoda aranea*	1	0.05
*Sturnira luisi* (1)	*Megistopoda proxima*	1	1.00
*Trachops cirrhosus* (4)	Speiseria ambigua*	2	0.04
	*Trichobius dugesioides*	20	1.00

* = non-primary association.

**Figure 4:**
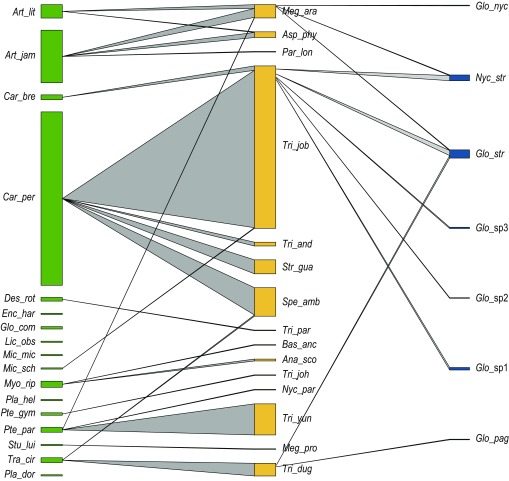
Interaction web between bat species (left), bat fly species (middle), and Laboulbeniales species (right) from Chucantí. The width of bars in each network level is proportional to the number of individuals.

*Trichobius joblingi* was the most common species (58.58%, *n* = 256), followed by *Trichobius yunkeri* (11.21%, *n* = 49), and *Speiseria ambigua* (10.30%, *n* = 45). Six species were represented by a single individual: *Megistopoda proxima*, *B. anceps, Nycterophilia parnelli*, *Paratrichobius longicrus, T. johnsonae*, and *T. parasiticus*. Overall, the collected bat flies presented high host specificity with 17 of 21 associations and 97.5% of all individual bat flies counted as primary associations; only occasionally bat flies were found on non-primary hosts (Figure 4; Table 3). Two bat fly species, *Aspidoptera phyllostomatis* and *Megistopoda aranea*, appeared to be sharing two bat host species as both were commonly collected from the two large *Artibeus* species captured in this study.

We found infection with Laboulbeniales on 30 of 437 bat flies (6.86%). All Laboulbeniales belonged to the genera *Gloeandromyces* and *Nycteromyces*. We found all 4 described species from neotropical bat flies: *G. nycteribiidarum*, *G. pageanus*, *G. streblae*, and *N. streblidinus*. In addition, based on both morphological characters and molecular data, we discovered 3 undescribed species in the genus *Gloeandromyces*. All species of Laboulbeniales encountered in Chucantí and their hosts are given in Table 4. Data on the prevalence of Laboulbeniales infection among bat fly species are presented in Table 5. The most frequently encountered species was *G. streblae* (on 12 bat flies of 3 species), followed by *N. streblidinus* (on 9 bat flies of 2 species). The thalli removed from five bat flies (1 *Speiseria ambigua*, 4 *T. joblingi*) were too young for secure identification to species level or even genus level.

**Table 4:** The species of Laboulbeniales found in Chucantí, with their bat fly hosts and the bat species from which the bat fly hosts were collected. Between parentheses is the number of bat flies observed with thalli of a given species, and the number of bat hosts if infected bat flies originated from different bat species. On some bat flies, only immature thalli were found, which were impossible to identify to species level (on 4 flies) or even genus level (on a single fly). These identifications are presented as sp. indet.

Laboulbeniales species	Bat fly host species	Bat host species
*Gloeandromyces nycteribiidarum*	*Megistopoda aranea* (1)	*Artibeus jamaicensis*
*Gloeandromyces pageanus*	*Trichobius dugesioides* (1)	*Trachops cirrhosus*
*Gloeandromyces streblae*	*Megistopoda aranea* (1)	*Artibeus jamaicensis*
	*Trichobius dugesioides* (4)	*Trachops cirrhosus*
	*Trichobius joblingi* (8)	*Carollia perspicillata*
*Gloeandromyces* sp. nov. 1	*Trichobius joblingi* (4)	*Carollia brevicauda* (1)
		*Carollia perspicillata* (3)
*Gloeandromyces* sp. nov. 2	*Trichobius joblingi* (1)	*Carollia perspicillata*
*Gloeandromyces* sp. nov. 3	*Trichobius joblingi* (2)	*Carollia perspicillata*
*Gloeandromyces* sp. indet.	*Trichobius joblingi* (3)	*Carollia perspicillata*
	*Speiseria ambigua* (1)	*Carollia perspicillata*
*Nycteromyces streblidinus*	*Megistopoda aranea* (1)	*Artibeus jamaicensis*
	*Trichobius joblingi* (8)	*Carollia brevicauda* (1)
		*Carollia perspicillata* (7)
Laboulbeniales sp. indet.	*Trichobius joblingi* (1)	*Carollia perspicillata*

**Table 5:** Overview of studied bat flies. Bat fly species sampled from Chucantí during this study, with the prevalence of Laboulbeniales infections and indication of parasite species.

Bat fly species	Bat host	No. sampled	No. infected	% infected	Laboulbeniales species
*Anatrichobius scorzai*	All	3	0		
*Aspidoptera phyllostomatis*	All	9	0		
*Basilia anceps*	*Myotis riparius*	1	0		
*Megistopoda aranea*	*Artibeus jamaicensis*	12	3	25.00	(1) *Gloeandromyces* *nycteribiidarum* (1) *Gloeandromyces streblae* (1) *Nycteromyces streblidinus*
*Megistopoda aranea*	Other bat host species	9	0		
*Megistopoda proxima*	*Sturnira luisi*	1	0		
*Nycterophilia parnelli*	*Carollia perspicillata*	1	0		
*Paratrichobius longicrus*	*Artibeus jamaicensis*	1	0		
*Speiseria ambigua*	*Carollia perspicillata*	42	1	2.38	*Gloeandromyces* sp.
*Speiseria ambigua*	Other bat host species	3	0		
*Strebla guajiro*	*Carollia perspicillata*	22	0		
*Trichobius anducei*	*Carollia perspicillata*	6	0		
*Trichobius dugesioides*	*Trachops cirrhosus*	19	5	26.32	(1) *Gloeandromyces pageanus* (4) *Gloeandromyces streblae*
*Trichobius dugesioides*	*Myotis riparius*	1	0		
*Trichobius joblingi*	*Carollia brevicauda*	6	1	16.67	*Gloeandromyces* sp. nov. 1 + *Nycteromyces streblidinus*
*Trichobius joblingi*	*Carollia perspicillata*	244	20	8.20	(2) *Gloeandromyces* sp. indet. (1) *Gloeandromyces* sp. indet. + *Nycteromyces streblidinus* (2) *Gloeandromyces* sp. nov. 1 (1) *Gloeandromyces* sp. nov. 1 + *Gloeandromyces streblae* (2) *Gloeandromyces* sp. nov. 3 (1) *Gloeandromyces* sp. nov. 2 (4) *Gloeandromyces* streblae (3) *Gloeandromyces streblae* + *Nycteromyces streblidinus* (1) Laboulbeniales sp. indet. (3) *Nycteromyces streblidinus*
*Trichobius joblingi*	Other bat host species	6	0		
*Trichobius johnsonae*	*Pteronotus gymnonotus*	1	0		
*Trichobius parasiticus*	*Desmodus rotundus*	1	0		
*Trichobius yunkeri*	*Pteronotus parnellii*	49	0		
Total		437	30	6.86	

*Trichobius joblingi* was most often infected with Laboulbeniales; of the 30 bat flies infected with Laboulbeniales, 21 were *T. joblingi*. This bat fly species also bore most diversity of Laboulbeniales. We found 5 species on this bat fly: *G. streblae*, 3 undescribed species of *Gloeandromyces*, and *N. streblidinus*. Finally, we observed double infections of Laboulbeniales, but only on *T. joblingi* bat flies. The following combinations of fungi occur together on a single host specimen: *G. streblae* with *G.* sp. nov. 1, *G. streblae* with *N. streblidinus*, and *G.* sp. nov. 1 with *N. streblidinus*. *Gloeandromyces* sp. nov. 1 was only observed at the base of the right wing, while the other two species occur on different positions of the host’s body.

### Molecular work

**Figure 5:**
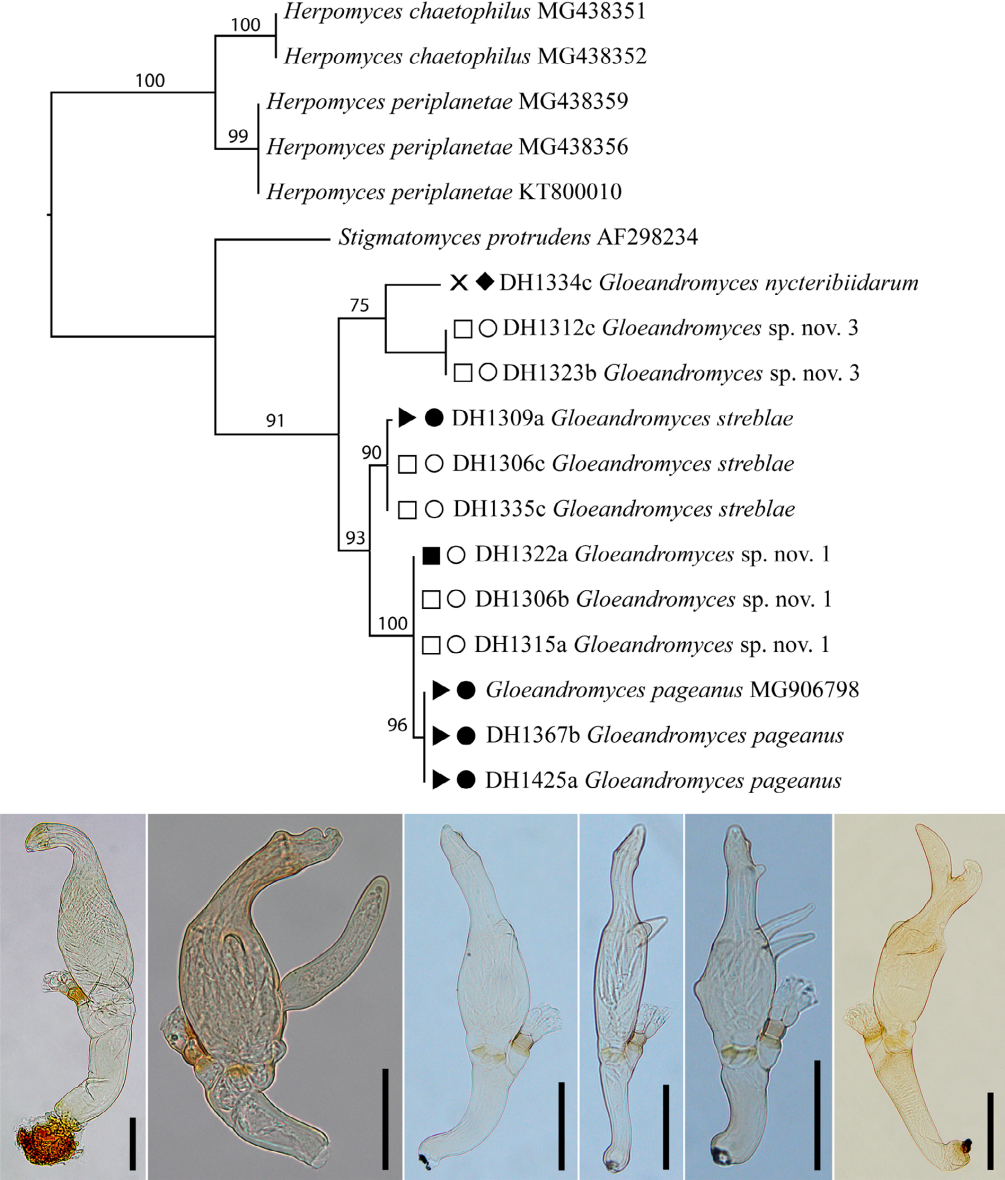
Phylogeny of the genus *Gloeandromyces*, with *Herpomyces* species as outgroup taxa, reconstructed from the LSU dataset. The topology is the result of maximum likelihood inference performed using Paup. For each node, the bootstrap support value (if > 70) is presented above the branch leading to that node. Bat hosts and bat fly hosts are indicated left of each isolate: □ *Carollia perspicillata*, ■ *C. brevicauda*, × *Artibeus jamaicensis*, ► *Trachops cirrhosus*; ● *Trichobius dugesioides*, ○ *T. joblingi*, ♦ *Megistopoda aranea*. Photos of thalli from left to right: *Gloeandromyces nycteribiidarum*, *G.* sp. nov. 3, *G.* streblae, *G.* sp. nov. 1, *G. pageanus*, and *G.* sp. nov. 2 (not present in phylogenetic reconstruction). Scale bars = 50 µm.

Our LSU dataset comprised 961 characters, of which 711 were constant and 231 were parsimony-informative. A total of 18 sequences were included in the dataset, of which 11 were newly generated during the course of this study, complemented by 7 sequences that we retrieved from GenBank: *Herpomyces chaetophilus* (2 isolates), *H. periplanetae* (3) as outgroup taxa; *Stigmatomyces protrudens* (1) (associated with Diptera: Ephydridae); *G. nycteribiidarum* (1), *G. pageanus* (3), *G.* sp. nov. 1 (3), *G.* sp. nov. 3 (2), and *G. streblae* (3). The genus *Gloeandromyces* was strongly supported in our ML phylogenetic reconstruction (Figure 5). All five species of the genus were supported by BS ≥ 75, usually even by BS ≥ 90.

*Gloeandromyces* sp. nov. 2 was found on a single *T. joblingi* bat fly only, only three thalli were present. We tried a DNA extraction using a single thallus as starting material, which was unsuccessful. However, based on morphological characters, we assume that these thalli represent an undescribed species. It is easily recognized by the perithecial neck carrying a large preapical projection, and a sigmoidal habitus. The same species was also collected in 2016 in Gamboa, Panama, again on a single *T. joblingi* bat fly (D. Haelewaters, unpublished data). No DNA extraction was attempted at that time.

## Discussion

### Bat ectoparasites and hyperparasites from Chucantí

**Figure 6:**
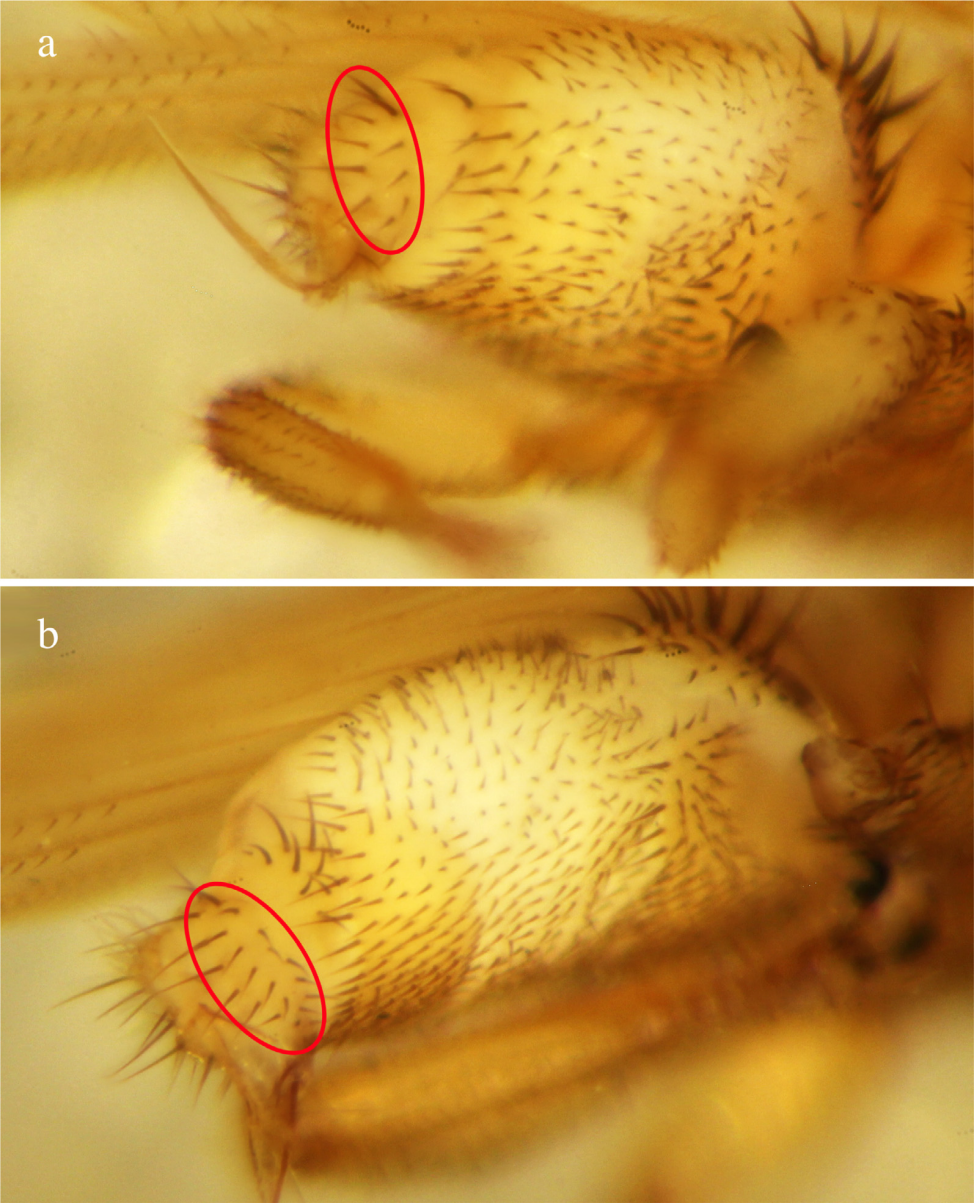
The abdomen of two male bat fly specimens, showing and comparing the typical setation of the 9th tergite in two *Trichobius* species [*sensu* 36]. (a) *Trichobius anducei* with 9–11 setae. (b) *Trichobius dugesioides* with 17–20 setae on the 9th tergite.

Our species list of ectoparasitic bat flies includes 16 species of which one, *T. anducei,* was previously unreported for Panama. With this report, the number of Panamanian bat fly species rises to 74 (Table 6). *Trichobius anducei* was described relatively recently [36] from *C. perspicillata* in Venezuela and has since been reported in Brazil [58] and Ecuador [80]. *Trichobius anducei* has been confused with *T. dugesioides* in previous studies; for this reason, Guerrero [36] presented distinct morphological characteristics for alcohol-preserved specimens, such as differences in setation of the 9th tergite in male bat flies, on which we based our identifications (see Figure 6). For Belize, ter Hofstede *et al.* [84] reported an unidentified bat fly from *C. perspicillata* and *C. brevicauda*, which the authors recognized to be similar to *T. dugesioides* and *T. anducei* and, together with unpublished records from Panama and Costa Rica (T. Hiller, unpublished data), might confirm the presence of *T. anducei* in Central America. Five bat fly species collected at Chucantí represent first reports for Darién. These are *B. anceps* (Nycteribiidae), *A. scorzai*, *N. parnelli*, *T. johnsonae*, and *T. parasiticus* (Streblidae). We are confident that more bat fly species are present at Chucantí for two reasons: the sample size for some bat species was small and certain bat host species known to occur in Darién [94] were not captured. Nevertheless, our new reports for Panama and the Darién province highlight the need for more taxonomical and ecological studies focusing on host-parasite interactions.

**Table 6:** All species of bat flies reported in Panama to date. Bat hosts reported in Darién are provided. Bat flies in bold are new reports for Darién. One of these, *Trichobius anducei*, also represents a new country record.

Bat fly species	Bat hosts in Darién	Reference(s)
NYCTERIBIIDAE		
* **Basilia anceps***	*Myotis riparius*	[39, this study]
* Basilia dunni*	*Myotis albescens*	[39]
* Basilia ferruginea*		[39]
* Basilia handleyi*		[39]
* Basilia myotis*	*Myotis nigricans*	[39]
* Basilia tiptoni*		[39]
* Basilia wenzeli*		[39]
STREBLIDAE		
* Anastrebla mattadeni*	*Anoura cultrata*	[94]
* Anastrebla modestini*		[94]
* Anastrebla nycteridis*	*Lonchophylla robusta*	[94]
* **Anatrichobius scorzai***	*Myotis riparius*	[94, this study]
* Aspidoptera phyllostomatis*	*Artibeus jamaicensis*	[27,94, this study]
	Artibeus lituratus*	[94, this study]
	Phyllostomus hastatus*	[27]
	Vampyressa nymphaea*	[94]
* Aspidoptera delatorrei*	*Sturnira lilium*	[27,94]
* Eldunnia breviceps*		[94]
* Exastinion clovisi*		[94]
* Joblingia schmidti*		[94]
* Mastoptera guimaraesi*	*Phyllostomus hastatus*	[27,94]
* Mastoptera minuta*	*Lophostoma silvicolum*	[94]
* Megistopoda aranea*	*Artibeus jamaicensis*	[27,94, this study]
	Artibeus lituratus*	[94, this study]
	Phyllostomus discolor*	[94]
	Pteronotus parnellii*	this study
* Megistopoda proxima*	*Sturnira lilium*	[94]
	*Sturnira luisi*	this study
* Megistopoda theodori*	*Sturnira ludovici*	[94]
* Metelasmus pseudopterus*	*Artibeus jamaicensis*	[27,94]
	Carollia perspicillata*	[94]
	Vampyressa nymphaea*	[94]
* Neotrichobius stenopterus*	*Dermanura phaeotis*	[27]
	Vampyressa pusilla*	[94]
* Noctiliostrebla maai*	*Noctilio albiventris*	[94]
* Noctiliostrebla traubi*	*Noctilio leporinus*	[94]
* Nycterophilia fairchildi*		[94]
* Nycterophilia natali*		[94]
* **Nycterophilia parnelli***	*Pteronotus parnellii*	[94, this study]
* Paradyschiria lineata*		[94]
* Paradyschiria parvuloides*	*Noctilio albiventris*	[94]
* Parastrebla handleyi*		[94]
* Paratrichobius dunni*	*Uroderma bilobatum*	[27,94]
* Paratrichobius longicrus*	Artibeus jamaicensis*	[94, this study]
* Paratrichobius lowei*	*Dermanura watsoni*	[94]
* Paratrichobius salvini*	*Chiroderma salvini*	[27,94]
* Paratrichobius sanchezi*	*Enchisthenes hartii*	[94]
* Paratrichobius* sp. (*longicrus complex*)	*Platyrrhinus vittatus*	[94]
* Pseudostrebla greenwelli*		[94]
* Pseudostrebla ribeiroi*		[94]
* Speiseria ambigua*	*Carollia castanea*	[27,94]
	*Carollia perspicillata*	[27,94, this study]
	Trachops cirrhosus*	this study
* Strebla altmani*	*Macrophyllum macrophyllum*	[94]
* Strebla alvarezi*		[94]
* Strebla guajiro*	*Carollia castanea*	[27,94]
	*Carollia perspicillata*	[27,94, this study]
* Strebla christinae*		[94]
* Strebla diaemi*		[94]
* Strebla galindoi*		[94]
* Strebla hertigi*	*Phyllostomus discolor*	[94]
* Strebla hoogstraali*		[94]
* Strebla kohlsi*	*Lophostoma silvicolum*	[94]
* Strebla mirabilis*	*Phyllostomus hastatus*	[27,94]
* Strebla wiedemanni*	*Desmodus rotundus*	[94]
* Trichobioides perspicillatus*	Sturnira lilium*	[94]
* **Trichobius anducei***	*Carollia perspicillata*	this study
* Trichobius bequarti*		[94]
* Trichobius brennani*	*Sturnira ludovici*	[94]
* Trichobius costalimai*	*Phyllostomus discolor*	[94]
* Trichobius dugesii*	*Glossophaga soricina*	[94]
* Trichobius dugesioides*	*Carollia perspicillata*	[94]
	?*Tonatia* sp.	[94]
	*Trachops cirrhosus*	this study
* Trichobius dunni*	*Molossus bondae*	[94]
* Trichobius galei*		[94]
* Trichobius joblingi*	*Carollia brevicauda*	this study
	*Carollia castanea*	[27,94]
	*Carollia perspicillata*	[27,94, this study]
	*Carollia subrufa*	[94]
	*Carollia* sp.	[94]
	Micronycteris schmidtorum*	this study
* **Trichobius johnsonae***	*Pteronotus gymnonotus*	[94, this study]
* Trichobius keenani*		[94]
* Trichobius lionycteridis*		[94]
* Trichobius lonchophyllae*	*Lonchophylla robusta*	[94]
* Trichobius longipes*	*Phyllostomus hastatus*	[27,94]
* Trichobius macrophylli*		[94]
* Trichobius mendezi*		[94]
* **Trichobius parasiticus***	*Desmodus rotundus*	[94, this study]
* Trichobius sparsus*	Phyllostomus hastatus*	[27,94]
* Trichobius uniformis*		[94]
* Trichobius urodermae*	*Uroderma bilobatum*	[94]
* Trichobius vampyropis*		[94]
* Trichobius yunkeri*	*Pteronotus parnellii*	[94, this study]

* = non-primary association [*sensu* 15,94]. Bat names are presented without subspecies designation and have been updated following Simmons [79].

The reported bat fly–bat interactions were highly specific; 97.5% off all bat fly individuals collected were associated with their primary host, comparable to other studies from Panama, Venezuela, and Paraguay [15,93,94]. The occurrence of single bat fly individuals on non-primary hosts is most likely due to contamination in the net or while handling the bat [13]. In most of these cases, the specific parasite is very abundant on their primary host, which was captured together with the non-primary host during the same sampling night. Due to the high capture numbers at two sampling sites, we sometimes placed two individuals of the same bat species (mostly *Carollia*) in a single cotton bag. However, we did not focus on parasite prevalences of bat flies on their bat hosts, so even when cross-contamination occurred in the bag, this had no influence on our host-association data. In cases of re-using cotton bags before washing them, we made sure to flip them inside-out first. Taking into account that bat flies have a limited lifespan when they are separated from their bat hosts (∼12 hours [72]), cross-contamination between sampling nights is highly unlikely.

The most common bat fly collected in this study was *T. joblingi*. Its primary host *C. perspicillata* was abundant at every capture site. The single individual collected from *M. schmidtorum* is therefore most likely an accidental occurrence. The same applies for a single individual of *M. aranea* from *P. parnellii* and the two individuals of *S. ambigua*, a bat fly which is easily distressed and flies off [15], from *Trachops cirrhosus.* The specificity index of *T. joblingi* on *Carollia brevicauda* is very low (0.03), indicating a non-primary host association, but the low number of *C. brevicauda* captured as well as literature records suggest that this is a primary host association [93,94]. The same might also be true for the parasite associations of the two large-sized *Artibeus* species, *A. jamaicensis* and *A. lituratus*, both sharing *M. aranea* and *A. phyllostomatis,* which are typically associated with *A. jamaicensis*. The single individual of *Paratrichobius longicrus,* a typical parasite of *A. lituratus*, was collected from *A. jamaicensis.* These host-parasite relationships were not as expected but are also previously reported for Panama by Wenzel *et al.* [94]. A probable explanation might be the uncertain status of *A. intermedius* and the overlap of morphological characteristics given in the literature, which often made definitive species identification in the field difficult. The combination of unclear species limits and possible contaminations during sampling most likely explains this inconsistent host-parasite relationship.

Bat flies are the most conspicuous and therefore best investigated group of bat-associated ectoparasites in Panama. However, there are over 50 species of other ectoparasitic arthropods reported from Panama, all identified based on morphological characteristics alone [94]. This list includes mites in the families Dermanyssidae (10 species), Spinturnicidae (16), and Trombiculidae (22); ticks (8); fleas (4); and true bugs (2). Given the fact that this study [94] was done over 50 years ago, more species – in particular for the mites – are expected in future studies focusing on these groups, especially when incorporating contemporary molecular phylogenetic techniques.

The thalli of Laboulbeniales encountered on bat flies from Chucantí represented 7 species. Of these, *G. nycteribiidarum* represents a new country record for Panama. *Gloeandromyces pageanus*, *G. streblae,* and *N. streblidinus* are new reports for Darién. In addition, we discovered 3 undescribed species in the genus *Gloeandromyces*. Laboulbeniales of bat flies are rarely reported. For example, *G. nycteribiidarum* has only been previously reported in Grenada [86] and Costa Rica [42]. In fact, the study of Laboulbeniales fungi associated with bat flies has been largely neglected for the past 80 years. Until the records presented here and by Haelewaters *et al.* [42], only the type collections were known for *G. nycteribiidarum*, *G. streblae*, and *N. streblidinus*. The discovery of 3 new species in a limited study area only hints at the true diversity of Laboulbeniales on neotropical bat flies. Laboulbeniales on bat flies from temperate regions, on the other hand, are apparently rather species-poor [7,41].

### Sequence-based identification of Laboulbeniales ectoparasitic fungi

Using LSU sequence data for species delimitation is a new development in Laboulbeniales taxonomy. To date, species have been delimited by the internal transcribed spacer region (ITS, consisting of ITS1–5.8S–ITS2) of the ribosomal DNA. However, in general the ITS region is hard to amplify for species of Laboulbeniales, even with recommended primers. Thus, whenever species of Laboulbeniales in genera without sequence data are considered for molecular work, most of the “general” ITS primers may not work. The reason is that both the ITS1 and ITS2 spacers are rapidly evolving regions [69], with primer mismatches as a result. We have no idea of the extent of variability in the ITS rDNA in genera for which no ITS sequences exist to date, such as *Gloeandromyces* and *Nycteromyces*.

However, the LSU region is much easier to amplify in Laboulbeniales with commonly used primers such as LR0R/LR5. Based on the evaluation of LSU sequence data in the genera *Herpomyces* [40] and *Hesperomyces*, we found that this marker has high discriminating power, comparable to the ITS (D. Haelewaters, unpublished data). Those two factors combined (easy amplification and high identification power) make the LSU region a favorable marker over the ITS rDNA. As a result, we focused on generating LSU sequences for this study. We were able to generate sequences for all species of *Gloeandromyces* but one; we found strong support for all 3 described species and 2 undescribed ones. (Support for the third undescribed species comes from morphological study.)

### Bat species richness and abundance

Several published studies have focused on bat diversity in Panama [3,43,51,62,77], but only a few reports are available for bats captured in Darién [43,62], and no studies are available from Chucantí. After an assessment of the literature, we found that all bat species from Chucantí had been reported before in Panama and, with the exception of *M. schmidtorum*, also in Darién. In fact, *M. schmidtorum* was previously only reported in the province of Los Santos [43]. The specimen of *P. dorsalis* from Chucantí represents the westernmost report for this species, and only the second from Panama. Previous records were from Cerro Mali in Darién (as *Vampyrops aquilus*), Colombia, Ecuador, and (northern) Venezuela [88].

Species richness in Chucantí was highest in the family Phyllostomidae. The two most abundant species accounted for 81.5% of total captures (*C. perspicillata* 62.56%, *A. jamaicensis* 18.94%). Based on surveys in Cerro Batipa (Chiriquí), Samudio Jr. [77] also found that the family Phyllostomidae was most diverse. However, in his field site at 1000–1200 m a.s.l. (comparable to our sites), the 6 most abundant species were (in decreasing order): *Artibeus toltecus*, *Sturnira mordax*, *Platyrrhinus vittatus*, *Sturnira hondurensis*, *A. lituratus*, and *C. brevicauda*. Studies in central Panamanian lowlands, mainland, and island captures around the Barro Colorado Nature Monument, also identified *A. jamaicensis* and *A. lituratus* as the predominate species [51]. In central Panamanian coastlands, at 850 m in Capira, Araúz [3] found 22 species, 19 of which were Phyllostomidae. Abundant species included *A. jamaicensis*, *A. lituratus* and *C. perspicillata*. This indicates that our findings on dominant families and species are consistent with previous studies conducted in other parts of Panama.

We found one individual of the bat *S. luisi*. *Sturnira* (Phyllostomidae, Stenodermatinae) is the most diverse genus in its family, with 22 described species and one undescribed species [90]. Until recently, the taxonomy of the genus was poorly understood and primarily based on morphological characteristics. Early reports of different *Sturnira* species, including *S. luisi*, were confused with *S. lilium* [88]. However, at present 21 monophyletic species-level clades have been identified based on molecular characters from 5 gene regions [89,90]. *Sturnira luisi* is considered abundant at high elevations [43, as *S. ludovici*] and occurs throughout Costa Rica to Ecuador and northwest Peru [79], although the presence of this species in Colombia has not been verified. *Sturnira luisi* has been found at Cerro Mali (1433 m) and Tacarcuna Village in Darién at 594 m, as well as Cerro Punta, Chiriqui (1402–1615 m), and upper Rio Changena, Bocas del Toro (732–1524 m).

Both *P. helleri* [77] and *Myotis riparius* [43] are uncommon species, which is in accordance with our findings. Previously, *M. riparius* bats have been found in Darién in Boca de Rio Paya and Tacarcuna Village at 594 m, as well as Armila (San Bias), and Cerro Azul (Panama) at 609 m [43, as *M. simus riparius*]. Both *M. schmidtorum* and *L. obscura* are rare species. *Micronycteris schmidtorum* is found in deciduous forests, and has only been recorded in Panama at Guanico, Los Santos province [43]. Our capture of *M. schmidtorum* is a new report for Darién. We found *L. obscura* at 681 m in the tropical broadleaf forest, which is different from previous findings where *L.* *obscura* was found in evergreen forests and fruit groves, in Tacarcuna Village at 975 m (Darién), Armila (San Blas), Almirante, and upper Rio Changena at 732 m (Bocas del Toro).

Following the determination keys by Handley Jr. [44] and Timm and LaVal [87], we identified three species of *Artibeus* on site, *A. intermedius*, *A. jamaicensis*, and *A. lituratus*. However, discussion exists about the validity of *A. intermedius*. Morphological studies show differences in skull morphology between larger-sized *A. lituratus* and the smaller *A. intermedius* [60], but molecular phylogenetic studies based on mitochondrial genes [46,76] leave the taxonomy of these two taxa unresolved. In the phylogeny by Guerrero *et al.* [38], based on mitochondrial DNA, a well-supported clade of *A. lituratus* from South America also contained *A. intermedius* isolates from Costa Rica, Honduras, Mexico, and Panama. The authors concluded that *A. intermedius* should be treated as a junior synonym of *A. lituratus* [*sensu* 5,79].

This survey shows there are many species taking advantage of the forest types of Chucantí, at all three elevations and vegetation types. We mostly captured phyllostomid bats, which is not surprising given the fact that mistnets are most effective in targeting this family [59]. High-flying bats are generally underrepresented using low residing capture methods such as mistnets and harp traps [20,25]. A return to Chucantí with additional equipment (ultrasonic bat detectors/triple-high systems) would complete our current picture of bat species.

### What can bats tell us about forest regeneration?

Bats play an important role in forest restoration and are indicators of forest health. An increased prevalence of bats in regions impacted by deforestation indicates regenerative secondary succession [8,64], that is, native flora returning to a disturbed area. This occurrence is coupled with the notion that the regenerative success is supportive of speciose bat populations, which occupy various niches [50]. In turn, bats permit swift forest regeneration, providing key ecological roles including habitat building, seed dispersal and pest control [51,54,57]. In Panama, deforestation and disturbance is an ongoing issue [68].

In 1997, three quarters of Darién was forested [68]. However, prominent tree stands, including the commercial cativo (*Prioria copaifera*) and the less commercially suitable cuipo (*Cavanillesia platanifolia*), continued to be felled for timber or removed in slash-and-burn operations for agricultural land to support jobs and livelihoods of locals [28,68]. The area of forest dominated by cativo has decreased by half between 1987 and 1999 [1,47]. The majority of deforestation of both cativo and cuipo is for agricultural purposes alone [68]; between 1987 and 1997 edges of existing cultivars had expanded into forest and increased agricultural area by up to 75%. This has posed a considerable threat to Darién forests and, subsequently, to their bat populations. However, deforestation prevention and conservation efforts are being supported by government, private organizations, and private landowners. The Food and Agriculture Organization of the United Nations (FAO) reports that 90.4% of Panama’s forests are privately owned [22], the 6 km^2^ Chucantí Nature Reserve being a prime example [10,48]. Efforts in Chucantí have aimed to reverse habitat degradation and recover the original ecosystem. These efforts have included removing agricultural farm animals and allowing forests to encroach on land, which had previously been used for cattle ranching [48].

The ability to fly allows bats to adapt to changes in their environment and among landscapes [52], while differences in morphology, and niche and dietary preferences allow individual species to respond differently to environmental stressors [24,50,63]. Frugivorous species, such as *Carollia*, benefit in forests of secondary succession, granted their roosting or foraging areas are not subject to change [49]. Once abundant, *Carollia* and other frugivorous species can speed forest regeneration, as they readily disperse seeds [57]. The abundance of *Carollia* and *Artibeus* in our study is supportive of previous findings, suggesting the abundance of neotropical frugivore species can increase in response to regeneration of tropical forests [63]. It is also likely that they contribute to the occurrence of early successional vegetation [67]. Importantly, the abundance of these two species highlights the regenerative success of Chucantí.

Bat species composition and abundance in Chucantí is relatively comparable to that of undisturbed forests in other parts of Panama [77], which most likely helps regenerate the forest. Although these secondary forests can harbor many bat species, host assemblages will likely still differ from primary forests [4]. This study provides insight into the current successful regenerative efforts in Chucantí, with potential trophic cascades affecting other inhabiting species. Through continued management [*sensu* 91], adaptable bat species capable of providing ecosystem services [51,54,67] can be attracted to these regenerating forests and speed up this secondary succession, further promoting the rehabilitation of the Chucantí Nature Reserve.

## Conclusions

This study shows that the relatively unexplored Darién province in Panama holds much to discover. During 68 mistnet hours, we captured bats representing 17 different species, of which *Micronycteris schmidtorum* was previously unreported in Darién and *Platyrrhinus dorsalis* represents the westernmost report for this species thus far. We screened captured bats for presence of ectoparasitic bat flies, which we in turn screened for presence of ectoparasitic Laboulbeniales fungi. The bat fly *Trichobius anducei* was sampled for the first time in Panama and five bat fly species are new reports for Darién. Finally, we found 7 species of Laboulbeniales on 30 bat flies, of which 1 species represents a new country record and 3 are undescribed. Considering the small sample size, we are confident that many more new discoveries will be made in this unique part of Panama.

## Conflict of interest

The authors declare that they have no conflicts of interest in relation to this article.

## Supplementary Material

Table S1 List of bat species captured at Chucantí Nature Reserve in Darién province, Panama. For each species, number of captured individuals (N), average forearm length (in mm), and average body mass (in g) are provided. Recaptures are not included in sample sizes or averages. Photos: Danny Haelewaters and Annabel Dorrestein. The Supplementary Material is available at https://www.parasite-journal.org/10.1051/parasite/2018017/olm.

## References

[1] M. 1999. Panamá Informe Ambiental. Autoridad Nacional del Ambiente, Panamá.

[2] Angehr G, Christian, D. 2000 Distributional records from the highlands of the Serranía de Majé, an isolated mountain range in eastern Panama. Bulletin-British Ornithologists Club 120, 173–178.

[3] Araúz J. 2002 Los murciélagos del sendero panamá, parque nacional altos de Campana, Panamá. Tecnociencia, 4, 35–48.

[4] Barlow J, Gardner TA, Araujo IS, Ávila-Pires TC, Bonaldo AB, Costa JE, Esposito MC, Ferreira LV, Hawes J, Hernandez MIM, Hoogmoed MS, Leite RN, Lo-Man-Hung NF, Malcolm JR, Martins MB, Mestre LAM, Miranda-Santos R, Nunes-Gutjahr AL, Overal WL, Parry L, Peters SL, Ribeiro-Junior MA, da Silva MNF, da Silva Motta C, Peres CA. 2007. Quantifying the biodiversity value of tropical primary, secondary, and plantation forests. Proceedings of the National Academy of Sciences, 104, 18555–18560. 10.1073/pnas.0703333104 PMC2141815 18003934

[5] Barquez RM, Perez S, Miller B, Diaz M. 2015. *Artibeus lituratus*. The IUCN red list of threatened species 2015. Accessed October 6, 2017.

[6] Benjamin RK. 1971. Introduction and supplement to Roland Thaxter’s contribution towards a monograph of the Laboulbeniaceae. Bibliotheca Mycologica, 30, 1–155.

[7] Blackwell M. 1980 Incidence, host specificity, distribution, and morphological variation in *Arthrorhynchus nycteribiae* and *A. eucampsipodae* (Laboulbeniomycetes). Mycologia, 72, 143–158.

[8] Castro‐Luna AA, Sosa VJ, Castillo‐Campos G. 2007 Bat diversity and abundance associated with the degree of secondary succession in a tropical forest mosaic in south‐eastern Mexico. Animal Conservation, 10, 219–228.

[9] Churchill S. 2008. Australian Bats, Sydney, Allen and Unwin.

[10] Colegrove L. 2017. Land purchase in Panama helps protect a “sky island” of cloud forest for threatened amphibians. Accessed October 4, 2017. https://www.rainforesttrust.org/news/strategic-land-purchase-in-panama-protects-a-sky-island-of-cloud-forest-for-threatened-amphibians/

[11] Darriba D, Taboada GL, Doallo R, Posada D. 2012 jModelTest 2: more models, new heuristics and parallel computing. Nature methods, 9, 772. 10.1038/nmeth.2109 PMC4594756 22847109

[12] De Kesel A, Haelewaters D. 2014 *Laboulbenia slackensis* and *L. littoralis* sp. nov. (Ascomycota, Laboulbeniales), two sibling species as a result of ecological speciation. Mycologia, 106, 407–414. 24871602 10.3852/13-348

[13] Dick CW. 2007 High host specificity of obligate ectoparasites. Ecological Entomology, 32, 446–450.

[14] Dick CW. 2013. Review of the bat flies of Honduras, Central America (Diptera: Streblidae). Journal of Parasitology Research, 2013, article ID 437696. 10.1155/2013/437696 PMC3619636 23634295

[15] Dick CW, Gettinger D. 2005. A faunal survey of streblid flies (Diptera: Streblidae) associated with bats in Paraguay. Journal of Parasitology, 91, 1015–1024. 10.1645/GE-536R.1 16419742

[16] Dick CW, Patterson BD. 2006. Bat flies: obligate ectoparasites of bats, in Micromammals and macroparasites: from evolutionary ecology to management. Morand, S., Krasnov, B. R. & Poulin, R, Editors. Springer: Tokyo. p. 179–194.

[17] Dinerstein E, Olson DM, Graham DJ, Webster AL, Primm SA, Bookbinder MP, Ledec G. 1995. A conservation assessment of the terrestrial ecoregions of Latin America and the Caribbean. Washington, DC: World Wildlife Fund-US. The World Bank.

[18] Dittmar K, Porter ML, Murray S, Whiting MF. 2006. Molecular phylogenetic analysis of nycteribiid and streblid bat flies (Diptera: Brachycera, Calyptratae): implications for host associations and phylogeographic origins. Molecular Phylogenetics and Evolution, 38, 155–170. 10.1016/j.ympev.2005.06.008 16087354

[19] Dormann CF, Gruber B, Fründ J. 2008 Introducing the bipartite package: analysing ecological networks. R News, 8, 8–11.

[20] Duffy AM, Lumsden LF, Caddle CR, Chick RR, Newell GR. 2000 The efficacy of Anabat ultrasonic detectors and harp traps for surveying microchiropterans in south-eastern Australia. Acta Chiropterologica, 2, 127–144.

[21] Edgar RC. 2004 MUSCLE: multiple sequence alignment with high accuracy and high throughput. Nucleic Acids Research, 32, 1792–1797. 15034147 10.1093/nar/gkh340 PMC390337

[22] FAO. 2005. Forest Resources Assessment 2005– Global Tables. Ownership of forest and other wooded land 2000. Accessed October 25, 2017. http://www.fao.org/forestry/32034/en/

[23] FAO. 2015. Global Forest Resources Assessment Rome: UN Food and Agriculture Organization.

[24] Fenton MB, Acharya L, Audet D, Hickey MBC, Merriman C, Obrist MK, Syme DM, Adkins B. 1992 Phyllostomid bats (Chiroptera: Phyllostomidae) as indicators of habitat disruption in the Neotropics. Biotropica, 24, 440–446.

[25] Flaquer C, Torre I, Arrizabalaga A. 2007 Comparison of sampling methods for inventory of bat communities. Journal of Mammalogy, 88, 526–533.

[26] Freeman PW. 1988 Frugivorous and animalivorous bats (Microchiroptera): dental and cranial adaptations. Biological Journal of the Linnean Society, 33, 249–272.

[27] González DP, Santos AM, Miranda JR. 2004 Streblidae (Diptera: Pupipara) ectoparásitos de murciélagos, en las tierras bajas del Parque Nacional Darien, provincia de Darién, Panamá. Tecnociencias, 6, 1–12.

[28] Grauel WT. 2004. Ecology and management of wetland forests dominated by *Prioria copaifera* in Darien, Panama. Doctoral dissertation, University of Florida, Gainesville, Florida.

[29] Guerrero R. 1993. Catalogo de los Streblidae (Diptera: Pupipara) parasitos de murcielagos (Mammalia: Chiroptera) del Nuevo Mundo I. Clave para los géneros y Nycterophiliinae. Acta Biologica Venezuelica, 14, 61–75.

[30] Guerrero R. 1994a. Catalogo de los Streblidae (Diptera: Pupipara) parasitos de murcielagos (Mammalia: Chiroptera) del Nuevo Mundo II. Los grupos: pallidus, caecus, major, uniformis y longipes del género *Trichobius* (Gervais, 1844). Acta Biologica Venezuelica, 15, 1–18.

[31] Guerrero R. 1994b. Catalogo de los Streblidae (Diptera: Pupipara) parasitos de murcielagos (Mammalia: Chiroptera) del Nuevo Mundo IV. Trichobiinae con alas desarrolladas. Boletín de Entomología Venezolana, 9, 161–192.

[32] Guerrero R. 1995a. Catalogo de los Streblidae (Diptera: Pupipara) parasitos de murcielagos (Mammalia: Chiroptera) del Nuevo Mundo III. Los grupos: dugesii, dunni y phyllostomae del género *Trichobius* (Gervais, 1844). Acta Biologica Venezuelica, 15, 1–27.

[33] Guerrero R. 1995b. Catalogo de los Streblidae (Diptera: Pupipara) parasitos de murcielagos (Mammalia: Chiroptera) del Nuevo Mundo V. Trichobiinae con alas reducidas o ausentes y miscelaneos. Boletín de Entomología Venezolana, 10, 135–160.

[34] Guerrero R. 1996. Catálogo de los Streblidae (Diptera: Pupipara) parásitos de murciélagos (Mammalia: Chiroptera) del Nuevo Mundo VI. Streblinae. Acta Biologica Venezuelica, 16, 1–26.

[35] Guerrero R. 1997. Catálogo de los Streblidae (Diptera: Pupipara) parásitos de murciélagos (Mammalia: Chiroptera) del Nuevo Mundo VII. Lista de especies, hospedadores y países. Acta Biologica Venezuelica, 17, 9–24.

[36] Guerrero R. 1998a. Notes on Neotropical batflies (Diptera, Streblidae). I. The genus *Trichobius*, with description of two new species and one new subspecies from Venezuela. Acta Parasitologica, 43, 86–93.

[37] Guerrero R. 1998b. Notes on Neotropical bat flies (Diptera: Streblidae): II. Review of the genus *Xenotrichobius*. Acta Parasitologica, 43, 142–147.

[38] Guerrero JA, Ortega J, Gonzalez D, Maldonado JE, Lorenzo C, Espinoza E. 2008. Molecular phylogenetics and taxonomy of the fruit-eating bats of the genus *Artibeus* (Chiroptera: Phyllostomidae). Avances en el Estudio de los Mamiferos de México. Publicaciones Especiales, 2, 125–146.

[39] Guimarães LR. 1966. Nycteribiid batflies from Panama (Diptera: Nycteribiidae), in Ectoparasites of Panama. Wenzel RL, Tipton VJ, Editors. Field Museum of Natural History: Chicago. p. 393–404.

[40] Haelewaters D, Pfliegler WP, Gorczak M, Pfister DH. In review. Birth of an order: comprehensive phylogenetic study excludes *Herpomyces* (Fungi, Laboulbeniomycetes) from Laboulbeniales. Molecular Phylogenetics and Evolution. 10.1016/j.ympev.2019.01.007 30625361

[41] Haelewaters D, Pfliegler WP, Szentivanyi T, Foldvari M, Sandor AD, Barti L, Camacho JJ, Gort G, Estok P, Hiller T, Dick CW, Pfister DH. 2017a. Parasites of parasites of bats: Laboulbeniales (Fungi: Ascomycota) on bat flies (Diptera: Nycteribiidae) in central Europe. Parasites & Vectors, 10, 96. 10.1186/s13071-017-2022-y PMC5320862 28222795

[42] Haelewaters D, Verhaeghen SJC, Rios Gonzalez TA, Bernal Vega JA, Villarreal Saucedo RV. 2017b. New and interesting Laboulbeniales from Panama and neighboring areas. Nova Hedwigia, 105, 267–299.

[43] Handley Jr. CO. 1966. Checklist of the mammals of Panama, in Ectoparasites of Panama. Wenzel RL, Tipton VJ, Editors. Field Museum of Natural History: Chicago. p. 753–795.

[44] Handley Jr. CO. 1981. Key to the bats of the lowlands of Panama. Washington DC: National Museum of Natural History.

[45] Holdridge LR, Grenke WC, Hatheway WH, Liang T, Tosi JA. 1971. Forest environments in tropical life zones: a pilot study. Forest environments in tropical life zones: a pilot study. Oxford, UK: Pergamon Press.

[46] Hoofer SR, Solari S, Larsen PA, Bradley RD, Baker RJ. 2008. Phylogenetics of the fruit-eating bats (Phyllostomidae: Artibeina) inferred from mitochondrial DNA sequences. Occasional Papers, The Museum Texas Tech University, 277, 1–15.

[47] INRENARE. 1987. Situación General de los Bosques de Cativo y su Utilización. Instituton Nacional de Recursos Naturales Renovables, Panama.

[48] International Conservation Fund of Canada (ICFC). 2017. Land acquisition of Cerro Chucantí nature reserve, Panama. Accessed October 25, 2017. http://icfcanada.org/our-projects/projects/panama-cerro-chucanti

[49] Jones KE, Barlow KE, Vaughan N, Rodríguez-Durán A, Gannon MR. 2001 Short-term impacts of extreme environmental disturbance on the bats of Puerto Rico. Animal Conservation Forum, 4, 59–66.

[50] Jones G, Jacobs DS, Kunz TH, Willig MR, Racey PA. 2009 Carpe noctem: the importance of bats as bioindicators. Endangered Species Research, 8, 93–115.

[51] Kalka MB, Smith AR, Kalko EK. 2008 Bats limit arthropods and herbivory in a tropical forest. Science, 320, 71–71. 18388286 10.1126/science.1153352

[52] Kalko EKV, Handley CO, Handley D. 1996. Organization, diversity, and long-term dynamics of a neotropical bat community, in Long-term studies of vertebrate communities. Cody ML, Smallwood JA, Editors. Academic Press: San Diego. p. 503–553.

[53] Kunz TH, Lumsden LF. 2003. Ecology of cavity and foliage roosting bats, in Bat ecology. Kunz TH, Fenton MB, Editors. University of Chicago Press: Chicago. p. 3–89.

[54] Kunz TH, Braun De Torrez E, Bauer D, Lobova T, Fleming TH. 2011. Ecosystem services provided by bats. Annals of the New York Academy of Sciences, 1223, 1–38. 10.1111/j.1749-6632.2011.06004.x 21449963

[55] Laurance WF. 2008 Adopt a Forest. Biotropica, 40, 3–6.

[56] Linhares AX, Komeno CA. 2000. *Trichobius joblingi*, *Aspidoptera falcata*, and *Megistopoda proxima* (Diptera: Streblidae) parasitic on *Carollia perspicillata* and *Sturnira lillium* (Chiroptera: Phyllostomidae) in southeastern Brazil: Sex ratios, seasonality, host site preference, and effect of parasitism on the host. Journal of Parasitology, 86, 167–170. 10.1645/0022-3395(2000)086[0167:TJAFAM]2.0.CO;2 10701585

[57] Lopez JE, Vaughan C. 2004 Observations on the role of frugivorous bats as seed dispersers in Costa Rican secondary humid forests. Acta Chiropterologica, 6, 111–119.

[58] Lourenço EC, Almeida JC, Famadas KM. 2016 Richness of ectoparasitic flies (Diptera: Streblidae) of bats (Chiroptera) — a systematic review and meta-analysis of studies in Brazil. Parasitology Research, 115, 4379–4388. 27503189 10.1007/s00436-016-5223-y

[59] Macswiney MC, Clarke FM, Racey PA. 2008 What you see is not what you get: the role of ultrasonic detectors in increasing inventory completeness in Neotropical bat assemblages. Journal of Applied Ecology, 45, 1364–1371.

[60] Marchán-Rivadeneira MR, Larsen PA, Phillips CJ, Strauss RE, Baker RJ. 2012. On the association between environmental gradients and skull size variation in the great fruit-eating bat, *Artibeus lituratus* (Chiroptera: Phyllostomidae). Biological Journal of the Linnean Society, 105, 623–634.

[61] Martínez‐Garza C, Howe HF. 2003 Restoring tropical diversity: beating the time tax on species loss. Journal of Applied Ecology, 40, 423–429.

[62] Mclellan LJ. 1984. A morphometric analysis of *Carollia* (Chiroptera, Phyllostomidae). American Museum Novitates, 2791, 1–35.

[63] Meyer CFJ, Kalko EKV. 2008 Assemblage‐level responses of phyllostomid bats to tropical forest fragmentation: land‐bridge islands as a model system. Journal of Biogeography, 35, 1711–1726.

[64] Meyer CF, Struebig MJ, Willig MR. 2016. Responses of tropical bats to habitat fragmentation, logging, and deforestation, in Bats in the anthropocene: Conservation of bats in a changing world. Voigt CC, Kingston T, Editors. Springer International Publishing: Cambridge, Massachusetts. p. 63–103.

[65] Miller MA, Pfeiffer W, Schwartz T. 2010. Creating the CIPRES Science Gateway for inference of large phylogenetic trees. Proceedings of the Gateway Computing Environments Workshop (GCE), 14 Nov. 2010, New Orleans, Louisiana. p. 1–8.

[66] Miller J, Tschapka M. 2001. The bat flies of La Selva (Diptera: Nycteribiidae, Streblidae). Accessed November 4, 2017. http://www.biologie.uni-ulm.de/bio3/Batfly/index.html

[67] Muscarella R, Fleming TH. 2007 The role of frugivorous bats in tropical forest succession. Biological Reviews, 82, 573–590. 17944618 10.1111/j.1469-185X.2007.00026.x

[68] Nelson GC, Harris V, Stone SW, Barbier EB, Burgess JC. 2001 Deforestation, land use, and property rights: empirical evidence from Darien, Panama. Land Economics, 77, 187–205.

[69] Nilsson RH, Kristiansson E, Ryberg M, Hallenberg N, Larsson KH. 2008 Intraspecific ITS variability in the kingdom Fungi as expressed in the international sequence databases and its implications for molecular species identification. Evolutionary Bioinformatics, 4, 193–201. 10.4137/ebo.s653 PMC2614188 19204817

[70] Oksanen J, Blanchet FG, Kindt R, Legendre P, Minchin PR, O’hara RB, Simpson GL, Solymos P, Stevens MHH, Wagner H, Oksanen MJ. 2013. Package ’vegan’. Community ecology package, version, 2(9).

[71] Ortiz OO, Baldini RM, Berguido G, Croat TB. 2016 New species of *Anthurium* (Araceae) from Chucantí Nature Reserve, eastern Panama. Phytotaxa, 255, 47–56.

[72] Overal WL. 1980. Host-relations of the bat fly *Megistopoda aranea* (Diptera: Streblidae) in Panama. University of Kansas Science Bulletin, 52, 1–20.

[73] Palmeirim JM, Etherdige K. 1985. The influence of man-made trails on foraging by tropical frugivorous bats. Influencia de los senderos hechos por el hombre en el forrajeo de murciélagos frugívoros tropicales. Biotropica, 17, 82–83.

[74] Patterson BD, Dick CW, Dittmar K. 2007 Roosting habits of bats affect their parasitism by bat flies (Diptera: Streblidae). Journal of Tropical Ecology, 23, 177–189.

[75] R Core Team. 2013. R: a language and environment for statistical computing. Vienna, Austria: R Foundation for Statistical Computing. http://www.R-project.org.

[76] Redondo RAF, Brina LPS, Silva RF, Ditchfield AD, Santos FR. 2008 Molecular systematics of the genus *Artibeus* (Chiroptera: Phyllostomidae). Molecular Phylogenetics and Evolution, 49, 44–58. 18662791 10.1016/j.ympev.2008.07.001

[77] Samudio Jr. R. 2002. Patterns of diversity and ecology in Panamanian bats at two elevations. Doctoral dissertation, University of Florida, Gainesville, Florida.

[78] Samudio Jr. R, Pino JL. 2014. Historia de la Mastozoología en Panamá. Historia de La Mastozoología En Latinoamérica, Las Guayanas Y El Caribe. Editorial Murciélago Blanco y Asociación, Quito y México DF, 329–344.

[79] Simmons NB. 2005. Order Chiroptera, in Mammal species of the world: a taxonomic and geographic reference. Third edition. Wilson DE, Reeder DAM, Editors. Johns Hopkins Univ Press: Baltimore, Maryland. p. 312–529.

[80] Stamper E. 2012. Host Specificity of Ecuadorian bat flies (Diptera: Streblidae). Honors College Capstone Experience/Thesis Projects. Paper 358. Accessed October 17, 2017. http://digitalcommons.wku.edu/stu_hon_theses/358

[81] Suzán G, Armién A, Mills JN, Marcé E, Ceballos G, Ávila M, Salazar-Bravo J, Ruedas L, Armién B, Yates TL. 2008 Epidemiological considerations of rodent community composition in fragmented landscapes in Panama. Journal of Mammalogy, 89, 684–690.

[82] Svensson MS, Samudio R, Bearder SK, Nekaris K. 2010 Density estimates of Panamanian owl monkeys (*Aotus zonalis*) in three habitat types. American Journal of Primatology, 72, 187–192. 19852005 10.1002/ajp.20758

[83] Swofford DL. 1991. PAUP: Phylogenetic Analysis Using Parsimony, Version 3.1 Computer program distributed by the Illinois Natural History Survey, Champaign, Illinois.

[84] ter Hofstede HM, Fenton MB, Whitaker Jr JO. 2004. Host and host-site specificity of bat flies (Diptera: Streblidae and Nycteribiidae) on Neotropical bats (Chiroptera). Canadian Journal of Zoology, 82, 616–626.

[85] Thaxter R. 1924. Contribution toward a monograph of the Laboulbeniaceae III. Memoirs of the American Academy of Arts and Sciences, 14:309–426, Plates I–XII.

[86] Thaxter R. 1931. Contribution toward a monograph of the Laboulbeniaceae V. Memoirs of the American Academy of Arts and Sciences, 16, 1–435, Plates I–LX.

[87] Timm RM, LaVal RK. 1998. A field key to the bats of Costa Rica. Occasional Publication Series, University of Kansas Center of Latin American Studies, 22, 1–30.

[88] Velazco PM. 2005. Morphological phylogeny of the bat genus *Platyrrhinus* Saussure, 1860 (Chiroptera: Phyllostomidae) with the description of four new species. Fieldiana: Zoology, n.s., 105, 1–53.

[89] Velazco PM, Patterson BD. 2013. Diversification of the yellow-shouldered bats, genus *Sturnira* (Chiroptera, Phyllostomidae), in the New World tropics. Molecular Phylogenetics and Evolution, 68, 683–698. 10.1016/j.ympev.2013.04.016 23632030

[90] Velazco PM, Patterson BD. 2014. Two new species of yellow-shouldered bats, genus *Sturnira* Gray, 1842 (Chiroptera, Phyllostomidae) from Costa Rica, Panama and western Ecuador. ZooKeys, 402, 43–66. 10.3897/zookeys.402.7228 PMC4023253 24843262

[91] Vleut I, Levy-Tacher SI, de Boer WF, Galindo-González J, Vazquez LB. 2013. Tropical secondary forest management influences frugivorous bat composition, abundance and fruit consumption in Chiapas, Mexico. PloS One, 8, e77584. 10.1371/journal.pone.0077584 PMC3795674 24147029

[92] Weir A, Hammond PM. 1997 Laboulbeniales on beetles: Host utilization patterns and species richness of the parasites. Biodiversity and Conservation, 6, 701–719.

[93] Wenzel RL. 1976. The streblid batflies of Venezuela (Diptera: Streblidae). Brigham Young University Science Bulletin-Biological Series, 20, 1–177.

[94] Wenzel RL, Tipton VJ. 1966. Ectoparasites of Panama. Chicago: Field Museum of Natural History.

[95] Wibbelt G, Speck S, Field H. 2009. Methods for assessing diseases in bats, in Ecological and behavioral methods for the study of bats. Kunz THP, Editor. John Hopkins University Press: Baltimore, Maryland. p. 775–794.

[96] Wolfe BT, Dent DH, Deago J, Wishnie MH. 2015 Forest regeneration under *Tectona grandis* and *Terminalia amazonia* plantation stands managed for biodiversity conservation in western Panama. New Forests, 46, 157–165.

[97] Woods CL, Dewalt SJ. 2013 The conservation value of secondary forests for vascular epiphytes in Central Panama. Biotropica, 45, 119–127.

