# Supplementary material for: A tripartite survey of hyperparasitic fungi associated with ectoparasitic flies on bats (Mammalia: Chiroptera) in a neotropical cloud forest in Panama
Source: Parasite. 2018 Apr 10;25:19. doi: 10.1051/parasite/2018017 (PMC5892177; doi:10.1051/parasite/2018017)
Supplement: Supplementary file 1 — List of bat species captured at Chucantí Nature Reserve in Darién province, Panama. For each species, number of captured individuals (N), average forearm length (in mm), and average body mass (in g) are provided. Recaptures are not included in sample sizes or averages. Photos: Danny Haelewaters and Annabel Dorrestein. [file parasite-25-19-s1.pdf]

Table S1. List of bat species captured at Chucantí Nature Reserve in the Darién Province, Panama. For each species, number of captured individuals (N), average forearm length (in mm), and average body mass (in g) are provided. Recaptures are not included in sample sizes or averages. Photos: Danny Haelewaters and Annabel Dorrestein.

Artibeus jamaicensis	N = 43<br>Avg. forearm = 62.3 mm, *n* = 43<br>Avg. body mass = 47.9 g, *n* = 42	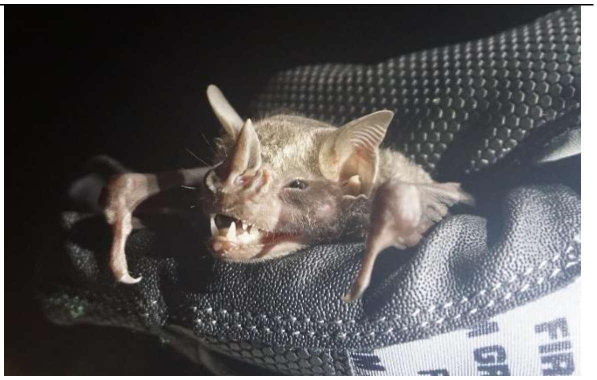 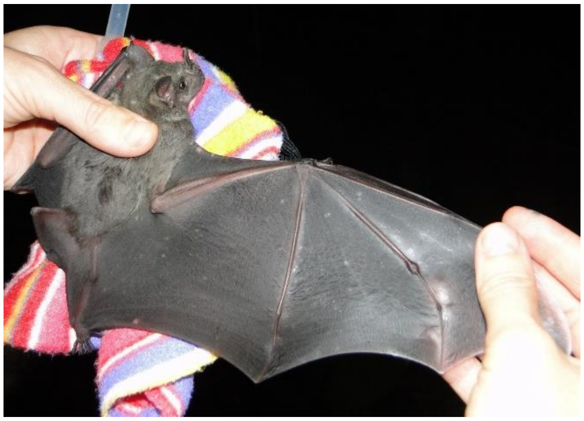
Artibeus lituratus	N = 11<br>Avg. forearm = 63.6 mm, *n* = 11<br>Avg. body mass = 45.9 g, *n* = 8	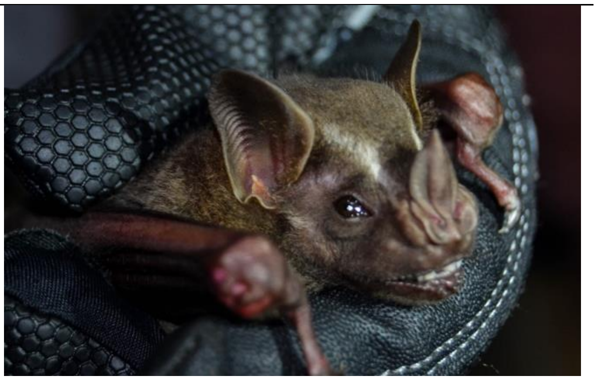 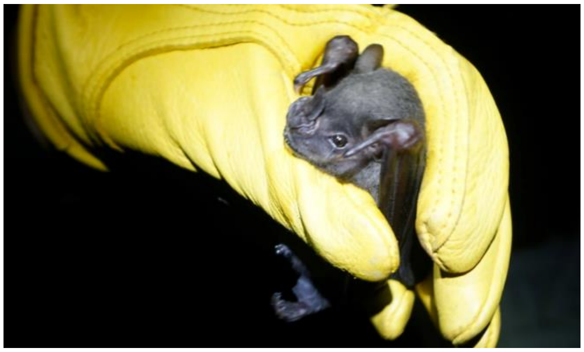

Carollia brevicauda	N = 4<br>Avg. forearm = 39.5 mm, *n* = 4<br>Avg. body mass = 18 g, *n* = 4	*Photos not available*
Carollia perspicillata	N = 142<br>Avg. forearm = 42.3 mm, *n* = 142<br>Avg. body mass = 20.8 g, *n* = 138	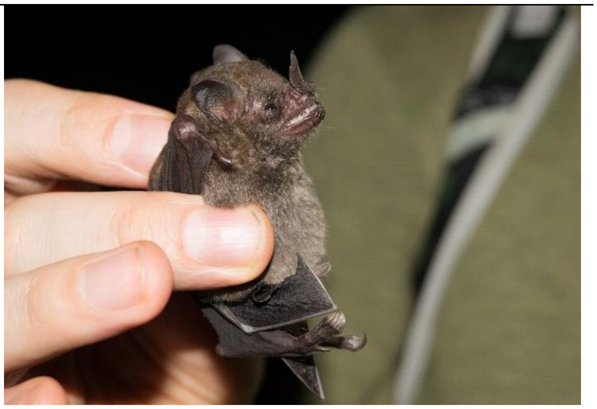<br>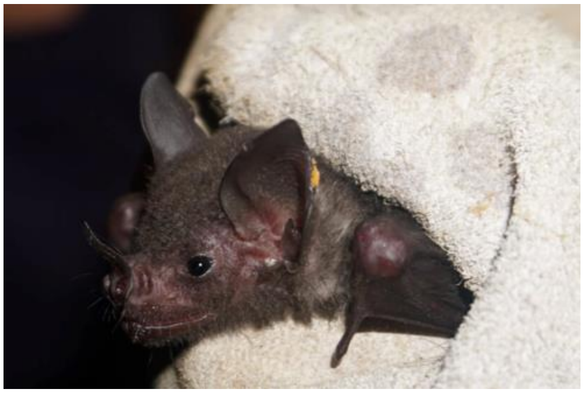
Desmodus rotundus	N = 2<br>Avg. forearm = 61.0 mm, *n* = 2<br>Avg. body mass = 41 g, *n* = 1	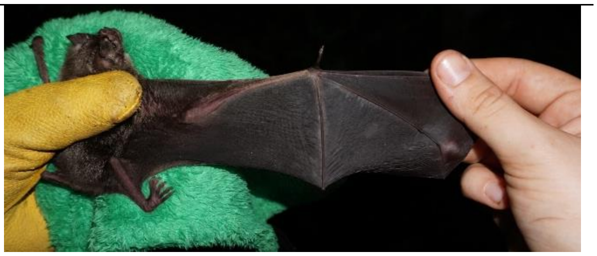<br>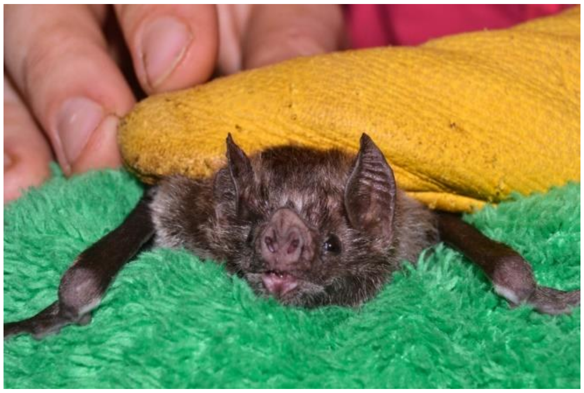

		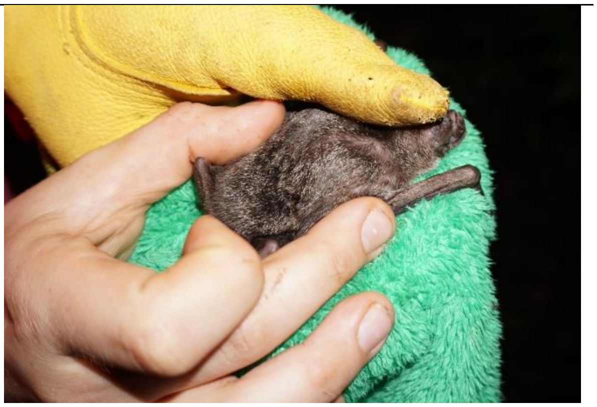
Enchisthenes hartii	N = 1<br>Avg. forearm = 42.3 mm, *n* = 1<br>Avg. body mass = 21 g, *n* = 1	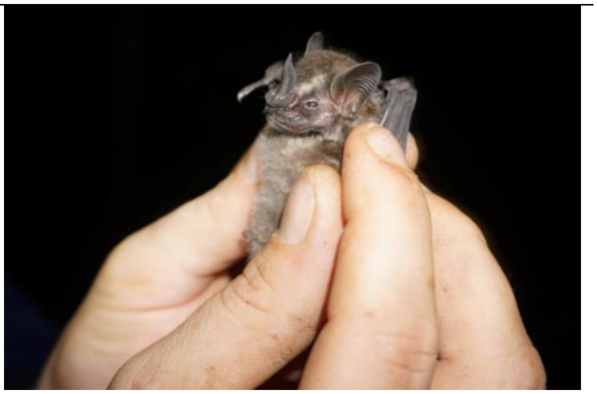
Glossophaga commissarisi	N = 2<br>Avg. forearm = 32.1 mm, *n* = 2<br>Avg. body mass = 8 g, *n* = 2	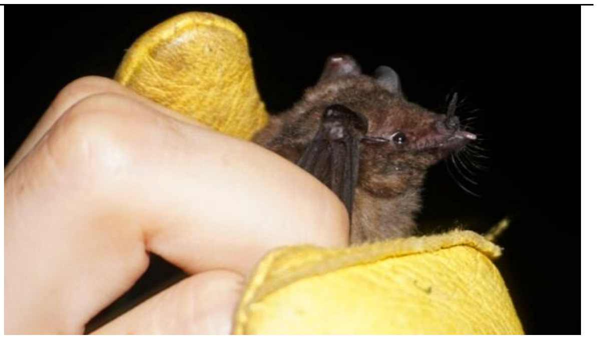
Lichonycteris obscura	N = 1<br>Avg. forearm = 33.2 mm, *n* = 1<br>Avg. body mass = n/a	*Photos not available*
Micronycteris microtis	N = 1<br>Avg. forearm = 34.2 mm, *n* = 1<br>Avg. body mass = 6 g, *n* = 1	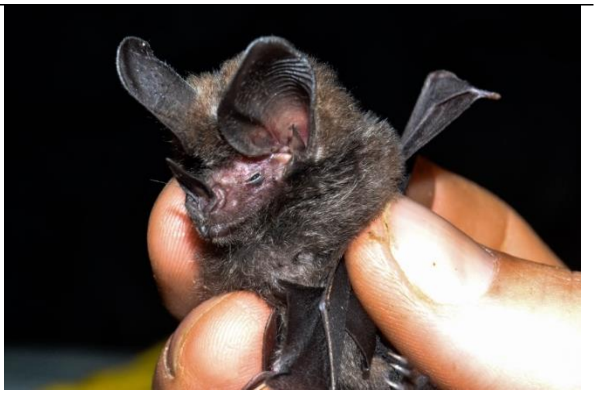

Micronycteris schmidtorum	N = 1<br>Avg. forearm = 33.8 mm, *n* = 1<br>Avg. body mass = n/a	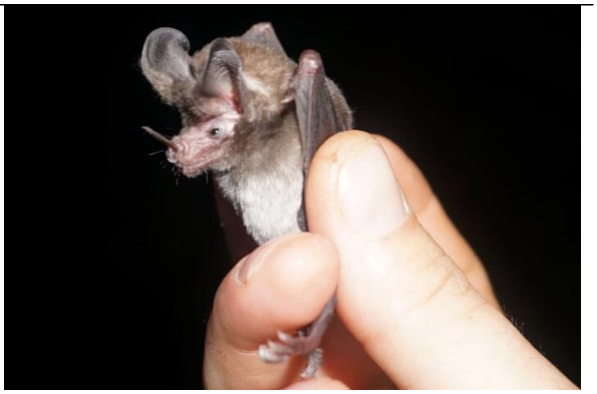
Myotis riparius	N = 5<br>Avg. forearm = 38.3 mm, *n* = 5<br>Avg. body mass = 5.6 g, *n* = 5	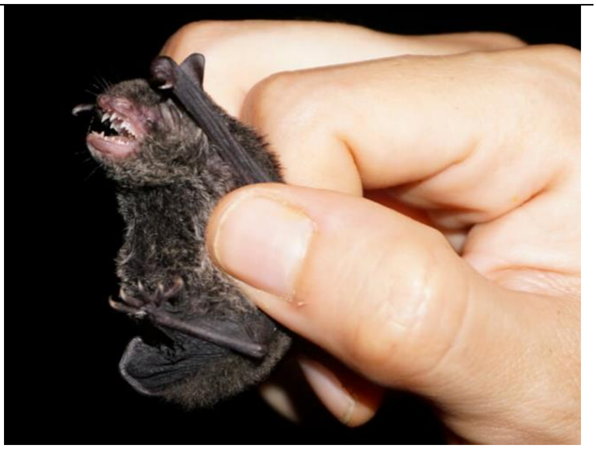<br>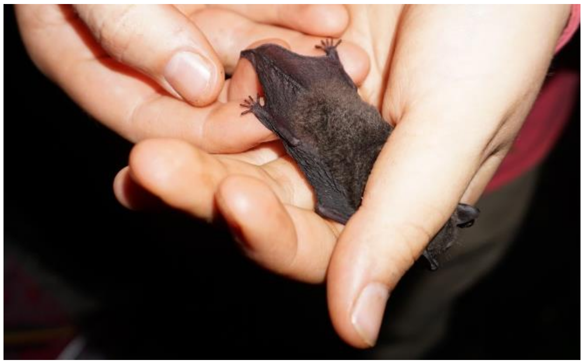<br>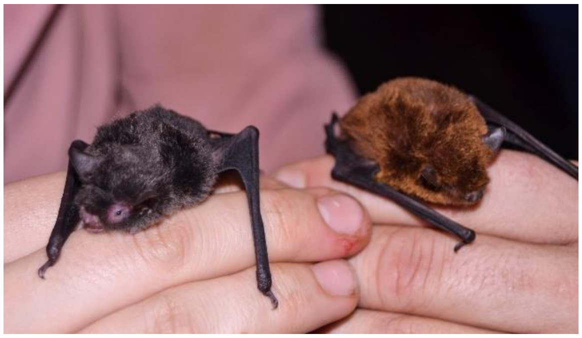

Platyrrhinus dorsalis	N = 1<br>Avg. forearm = 53.4 mm, n = 1<br>Avg. body mass = 51 g, n = 1	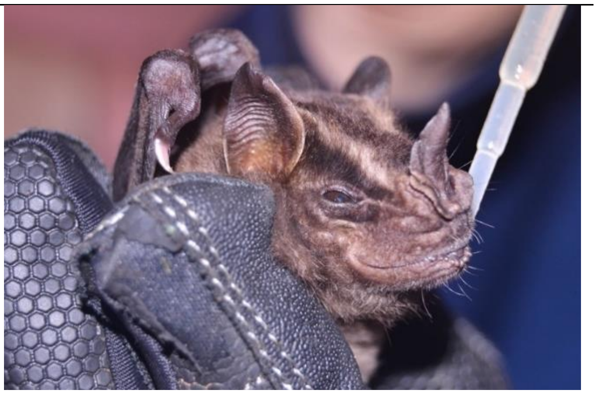<br>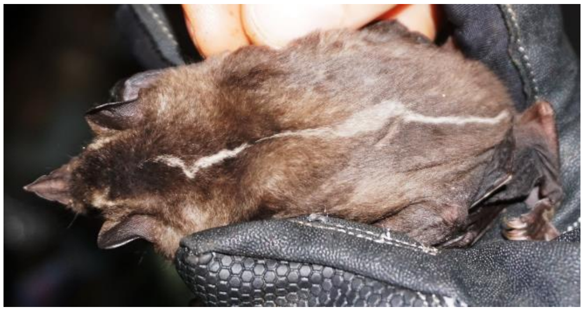
Platyrrhinus helleri	N = 1<br>Avg. forearm = 38.9 mm, *n* = 1<br>Avg. body mass = 33 g, *n* = 1	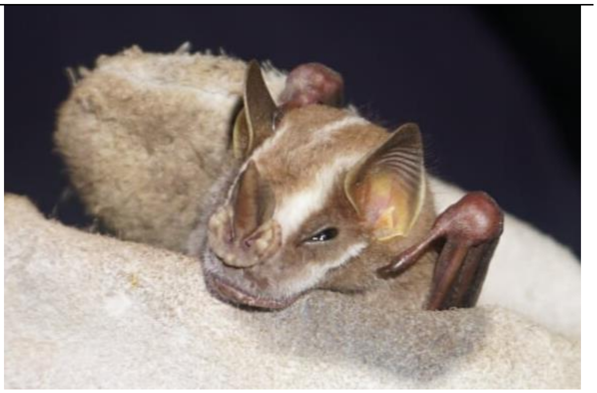<br>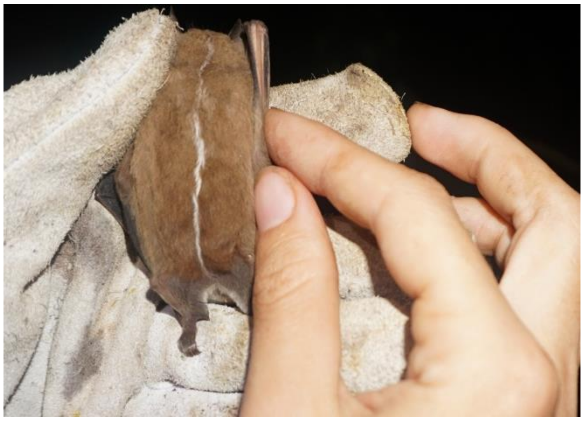

Pteronotus gymnonotus	N = 2<br>Avg. forearm = 53.3 mm, *n* = 2<br>Avg. body mass = 18 g, *n* = 2	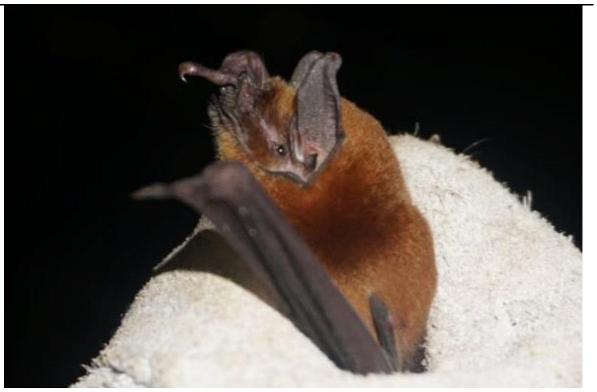<br>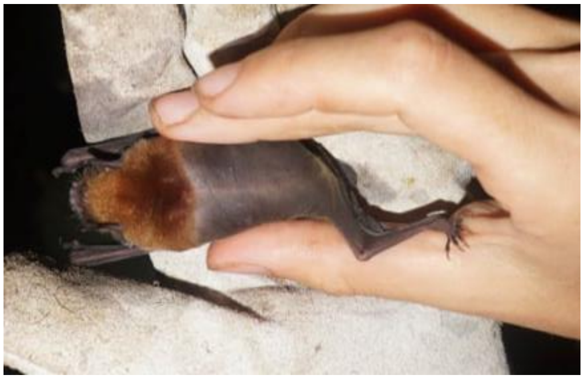<br>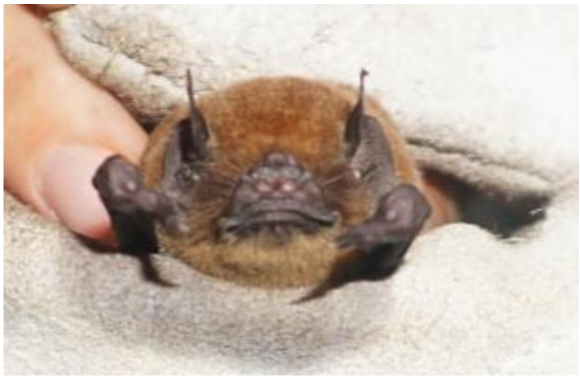
Pteronotus parnellii	N = 4<br>Avg. forearm = 61.3 mm, *n* = 4<br>Avg. body mass = 27.3 g, *n* = 4	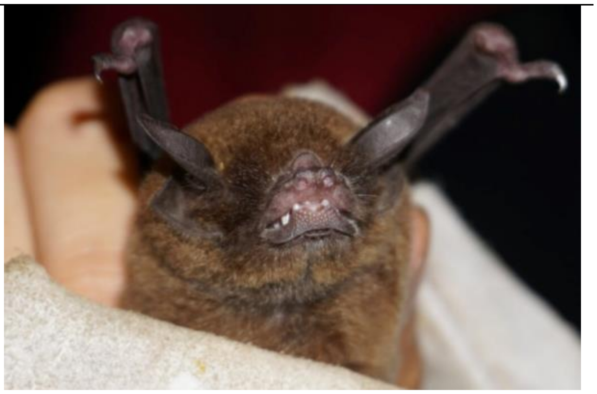

		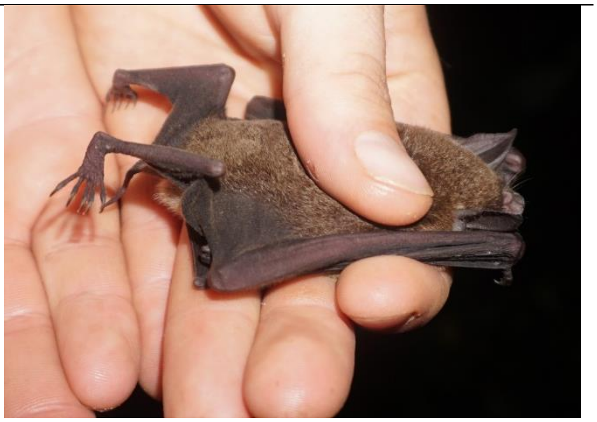
Sturnira luisi	N = 1<br>Avg. forearm = 43.1 mm, *n* = 1<br>Avg. body mass = 24 g, *n* = 1	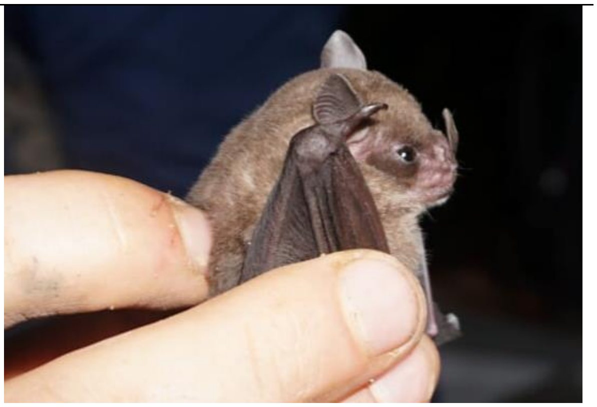<br>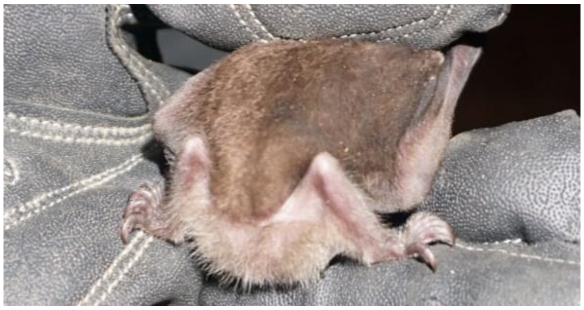<br>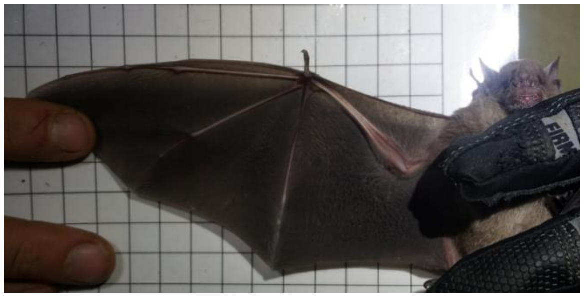
